# Life in the brine of Lunenburg, Germany: unveiling microorganisms associated with Zechstein salt deposits

**DOI:** 10.3389/fmicb.2025.1625916

**Published:** 2025-11-12

**Authors:** Katharina Runzheimer, Laura Schwab, Denise Engel, Christoph Schaudinn, Michael Laue, Katarína Rebrošová, Kristina Beblo-Vranesevic, Muhaiminatul Azizah, Stefan Leuko

**Affiliations:** 1 Department of Applied Aerospace Biology, Institute of Aerospace Medicine, German Aerospace Center (DLR e.V), Cologne, Germany; 2 Institute of Biodiversity, Ecology and Evolution, Friedrich Schiller University Jena, Jena, Germany; 3 Robert Koch Institute, Berlin, Germany; 4 Department of Microbiology, Masaryk University, Brno, Czechia; 5 Institute for Inorganic and Analytical Chemistry, Bioorganic Analytics, Friedrich Schiller University Jena, Jena, Germany

**Keywords:** Brine, Lunenburg, halophilic archaea, *Haloarcula*, *Halorubrum*, Zechstein Sea

## Abstract

**Introduction:**

The presence of hypersaline brines on other planets and moons in the inner and outer Solar System has been well established. Hence, any theory of life on other planets must consider microorganisms adapted to high salt concentrations. The hypersaline brine from Lunenburg (Germany) with 302.25 g L^−1^ NaCl, originating from the remnants of the Zechstein Sea, has long been utilized to harvest salt, but potential microbial life in the brine had never been investigated.

**Methods:**

We employed cultivation-based and -independent methods to characterize the microbial diversity, while also analyzing environmental parameters. Specifically, we performed V1/V2 and V3/V4 amplicon sequencing of environmental DNA and conducted haloarchaeal-focused cultivation and enrichments. Furthermore, we conducted whole-genome sequencing and analysis, Raman spectroscopy, electron and fluorescence microscopy, and compatible solute analysis on two isolates from the frequently cultivated genera *Haloarcula* and *Halorubrum*.

**Results:**

Our findings proved the presence of a broad range of halophilic microorganisms, including sulfate-reducing bacteria, haloarchaea and yet-uncultivated microorganisms like *Nanohaloarchaeota* and *Patescibacteria* in the Lunenburg brine. Two haloarchaeal isolates were described in more detail, revealing the presence of bacterioruberin for oxidative stress protection, potential polyhydroxyalkanoates for energy storage, pleomorphic structures as well as ‘package-like aggregates’ as possible adaptations to extreme conditions. Distinct osmotic adaptation strategies and a low average isoelectric point of the isolates’ proteomes were identified.

**Discussion:**

Our research shows that the hypersaline brine from Lunenburg harbors a diverse microbial community and is an ideal and easily accessible testbed to search for yet-uncultivated microorganisms as well as novel microorganisms to use for astrobiological studies.

## 1 Introduction

The search for evidence for extant or extinct life on other planets or moons in our solar system has fascinated humankind for decades, and new habitable planets and moons are discovered at an astonishing rate. At the moment, however, we cannot go there in person to search for life, no matter how big or small it may be, and the abilities of robots are limited. Therefore, the study of terrestrial analogues is highly important as to refine the limits of life. In this manuscript, we focus on halophilic archaea, as they are considered to be of high astrobiological interest, possessing an ancient evolutionary lineage, and exceptional physiological adaptability. They are also capable of thriving in diverse planetary environments, highlighting their importance for *in situ* life detection beyond Earth (DasSarma et al., 2020).

Evidence for liquid brines and salts were already found on Mars, Enceladus and Europa (Postberg et al., 2009; Trumbo et al., 2019; Carrier et al., 2020). In the ancient Noachian epochs (4.5–3.7 billion years ago) of Mars, water was plentiful across its surface. However, as time progressed, Mars underwent a transformation, evolving into the cold and arid world we observe today (Jakosky, 2021). Something similar, yet by far less drastic, happened during the late Permian on Earth. During this time, the European Southern Permian Basin covered roughly 600,000 km^2^ and lay beneath the epicontinental Zechstein Sea. It extended from the British Isles in the west, across Central Europe, including the North Sea, to the Latvian-Lithuanian and Belarusian-Polish border in the east (García-Veigas et al., 2011; Sendula et al., 2022).

The end-Permian event around 251 million years ago, Earth’s largest mass extinction of the past 600 million years, was triggered by a rapid rise in temperature, most likely caused by volcanic eruptions (Benton and Twitchett, 2003). In this time the Zechstein ocean evaporated and formed the Zechstein salts (Sendula et al., 2022). Throughout this process, following a series of marine transgressions and regressions, seven distinct cycles of Zechstein salt with unique composition and thickness were deposited during the Upper Permian, and subsequently layered with marine and terrestrial sediments [reviewed by Zhang et al. (2013)]. Salt behaves plastically under pressure, accumulating and rising to form salt walls and diapirs. While salt structures are common throughout northern Germany, typically hundreds of meters below the surface, they only reach the surface in three cities: Sperenberg, Bad Segeberg, and Lunenburg (Sirocko, 2012). In this study, we sampled and examined the Lunenburg brine located in northern Germany. Groundwater dissolves the salt deposits and can be harvested as a saturated salt brine above the ground. During the late Middle Ages, Lunenburg was among of Europe’s leading centers of salt production, evaporating brine from wells in large pans (Trüper, 2004). Nowadays, the brine can be accessed via a faucet at the German Salt Museum in Lunenburg and is directly pumped from the well.

Hypersaline ecosystems on Earth are generally associated with halophilic archaea, but bacteria and algae always co-occur in lower abundances. Bacteria are predominantly made up by the genus *Salinibacter*, which were reported to be prevalent in 5%–25% of the total prokaryotic community within crystallizer ponds from solar salterns (Antón et al., 2000), while the algae *Dunaliella* accounts for the majority of primary production in hypersaline environments worldwide (Oren, 2005). Typical environments for halophilic archaea are the Dead Sea (Bodaker et al., 2009), solar salterns (Boujelben et al., 2014), salt brine lakes in Antarctica (Williams et al., 2017; Le Lay et al., 2023), and the high-altitude Andes (Runzheimer et al., 2024), as well as arid environments (Bachran et al., 2019). However, they can also be found in lower-salinity hypersaline environments such as Shark Bay (Australia), where they are associated with stromatolites and microbial mats (Allen et al., 2008; Goh et al., 2009).

Some haloarchaeal species have even been recovered from halite as old as 225–280 million years (Stan-Lotter et al., 2002), raising the question of how they survived. Halophilic archaea are not known to form dormant bodies such as spores (Radax et al., 2001), however, phenotypic variants, known as persister cells, were reported (Megaw and Gilmore, 2017). They persist in halite and resist the desiccation probably due to being polyploid and using DNA as a phosphate storage polymer, formation of dwarfing cells, and dormant stages like spherical forms. They also produce extracellular polymeric substances and proteins like halomucein, which protects the cells by building a capsula around the cell (Stan-Lotter and Fendrihan, 2015). It was already hypothesized, that especially polyploidy may be a necessary condition for the persistence of this long period of time, due to the chemical instability of DNA (Soppa, 2015).

In terms of salinity, *Halobacteriales*, within the domain of archaea, is the order with the highest salt requirement and most of its genera need over 100–150 g L^–1^ salt for growth and survival. Strategies to survive this high dose involve the production or uptake of compatible solutes as well as accumulation of KCl in combination with an acidic proteome, which is the preferred method of haloarchaea and the reason beyond their obligate requirement on salt within the medium (Oren, 2008). The “salt-in” strategy is based on KCl rather than on NaCl, because Na^+^ ions are excluded as much as possible in all domains of life (Gunde-Cimerman et al., 2018), due to their inhibitory effect on enzymes in high concentrations (Brown and Simpson, 1972). Energy-consuming import of Cl^–^ ions is required alongside K^+^ ions to maintain osmotic balance (Gunde-Cimerman et al., 2018) and is, among others, carried out with the light-driven chloride pump halorhodopsin (Schobert and Lanyi, 1982). Na^+^/H^+^ antiporter systems, and active, as well as passive, K^+^ channels are further involved in maintaining the “salt-in” strategy. Required energy is delivered by respiratory electron transport, with the addition of the light dependent proton pump bacteriorhodopsin (Gunde-Cimerman et al., 2018; Straková et al., 2025). Compatible solutes were originally defined in 1972, and are solutes which do not negatively affect enzymes, even in high concentrations (Brown and Simpson, 1972). Among halophilic archaea, synthesis of trehalose, and uptake of glycine-betaine are universally widespread (Youssef et al., 2013). However, also trehalose/maltose transport systems in *Halorubrum* (Yang et al., 2022), and parts of the ectoine synthesis pathway for *Halogranum*, *Halalkalicoccus, Haloarcula*, and *Natrinema* (Kiledal et al., 2023; Straková et al., 2025) were reported. In addition, proline, choline and proline-betaine in *Natrinema* and *Haloarcula* species were detected within genomic analysis (Straková et al., 2025).

Next to compatible solute production and KCl accumulation, selective pressure led to highly acidic amino acid compositions within halophilic microorganisms to prevent “salting out” by hydration of the proteins’ shells, while maintaining enzymatic function in highly saline environments (Hartman et al., 2010). Usually, the isoelectric point (pI) of bacteria and archaea has a bimodal distribution around 5 for cytoplasmic proteins and 9 for membrane-bound proteins (Schwartz et al., 2001). However, halophilic archaea have adapted to high osmotic stress by proteome acidification. The major peak of protein pI values is around 4–5, with exceptions including transporters, certain ribosomal subunits, membrane-associated and DNA-binding proteins, and some hypothetical proteins (Hartman et al., 2010, Becker et al., 2014, Straková et al., 2025). Proteins with high pI are insulated from the hypersaline cytosols acidifying selection, because they are membrane-shielded or internally located (Becker et al., 2014).

Another potential important metabolite for halophilic archaea, is the carotenoid pigment bacterioruberin, which shows antioxidative activity and improves the capability to survive higher radiation doses [reviewed by Grivard et al., 2022]. The C_50_ carotenoid bacterioruberin is built primarily by halophilic archaea (Giani et al., 2024), but also by several cold-adapted bacteria (Flegler et al., 2020; Strand et al., 1997). For astrobiological studies, this pigment is not limited in terms of survival to radiation and persistence in halite. It also represents an interesting target and biomarker for life detection, using remote *in situ* Raman spectroscopy (Marshall et al., 2007). Additionally, to overcome starvation within oligotroph brines and halites, some microorganisms are capable to produce lipid carbon storage polymers, also referred to polyhydroxyalkanoate (PHA), polyhydroxybutyrate (PHB), and polyhydroxyvalerate (PHV) (Simó-Cabrera et al., 2021). These storage polymers were already reported for diverse haloarchaeal genera including *Haloferax* (Simó-Cabrera et al., 2024) and *Halolamina* (Hagagy et al., 2022). Taking all this information into account, halophilic archaea represent a promising, yet not fully discovered, microbial lineage to investigate for further research on extremophilic microorganisms and space exploration missions.

The Lunenburg hypersaline site had never been microbiologically characterized, leaving its microbial diversity and adaptation strategies unknown. Based on previous research from analogous hypersaline environments, we hypothesized that it harbors a community dominated by halophilic archaea. Their survival is expected to rely on physiological strategies such as compatible solutes and ion antiporters, together with genetic adaptations including pigments and polyhydroxyalkanoates. To test this hypothesis, we combined geochemical analyses with cultivation-independent and haloarchaeal enrichment approaches to determine osmotic pressure, available energy sources, and microbial diversity. Genomic sequencing, electron and fluorescence microscopy, and Raman spectroscopy of isolates were applied to uncover the mechanisms of microbial adaptation to this extreme environment.

## 2 Materials and methods

### 2.1 Sampling and hydrochemical analysis

The Lunenburg brine at the German Salt Museum was collected on three occasions (September 2022, October 2023, and April 2024), each with a distinct objective. Campaign 1 (pilot) investigated whether halophilic archaea could be cultivated from the brine and optimized the sampling as well as handling protocols. Campaign 2 (16S rRNA gene amplicon sequencing) profiled the microbial community independently of cultivability and helped identify present microbial taxa within the brine. Within campaign 3 (enrichment and parameters), key environmental parameters were quantified and enrichment experiments were performed using an expanded panel of culture media with differing compositions to assess the cultivability of a broader range of halophilic archaea.

The brine was accessed via a faucet at the Salt Museum, and was pumped from the well at a depth of approx. 40 m with a pipe distance of 100–150 m. In addition to prior analyses of the brine composition by the German Salt Museum (Table 1), we measured hydrochemical parameters including temperature, conductivity, salinity, total dissolved solids (TDS), as well as resistivity directly at the sampling site within three distinct measurements. The pH was measured twice (Metler Toledo, InLab 738-ISM-5 m, Inlab Expert Gp-5 m-ISM) (Table 2). Ca^2+^, K^+^, Mg^2+^, Na^+^, F^–^, Cl^–^, SO_4_^2–^, Br^–^, NO_3_^–^, NO_2_^–^ and the dissolved organic carbon (DOC) content were analyzed externally after particle filtration using a polyethersulfone filter with a pore size of 0.45 μm. The sample for major cation analysis (Ca^2+^, K^+^, Mg^2+^, Na^+^) was additionally acidified (pH 2) with nitric acid (68%), and measured using inductively coupled plasma atomic emission spectrometry, whilst anions (F^–^, Cl^–^, SO_4_^2–^, Br^–^, NO_3_^–^, NO_2_^–^) were quantified through ion chromatography. The concentrations were measured in triplicates. The DOC content was determined after high-temperature catalytic oxidation and non-dispersive infrared detection and was measured in duplicates (Lehmann et al., 2021).

**TABLE 1:** Components of the Lunenburg brine and the concentrated kettle pan salt with 0.01% H_2_O residue (Deutsches Salzmuseum [German Salt Museum], n.d.).

Solute (salt/mineral)	Brine	Kettle pan salt
Sodium chloride (NaCl)	302.25 g L^–1^	97.24%
Carnalite (KMgCL_3_)	7.94 g L^–1^	n.d.
Gypsum (CaSO_4_)	3.37 g L^–1^	0.54%
Epsom salt (MgSO_4_)	5.62 g L^–1^	1.00%
Soda (Na_2_CO_3_)	0.19 g L^–1^	0.03%
Iron oxide (FeO)	1.50 mg L^–1^	n.d.
Potassium chloride (KCl)	n.d.	0.78%
Magnesium chloride (MgCl_2_)	n.d.	0.40%

N.d., not determined.

**TABLE 2:** Environmental parameters of the Lunenburg brine.

Parameter	Value
Sample type	Water
Geographical location	53°14’38.8”N 10°24’07.3”E
Resistivity (Ωcm)	4.21 ± 0.05
pH	6.4
TDS (g L^–1^)	118.44 ± 1.67
Salinity (ppt)*	300.5 ± 15.22
Conductivity (mS cm^–1^)	236.67 ± 2.87
Temperature (°C)	15.55 ± 0.35
Dissolved organic carbon (mg L^–1^)	3.04-3.437 ± 0.147/0.394
Oxygen (O_2_) (mg L^–1^)	2.98
Chloride (Cl^–^) (g L^–1^)	177.6 ± 0.140
Sodium (Na^+^) (g L^–1^)	107.2 ± 0.152
Sulfate (SO_4_^2–^) (mg L^–1^)	9246 ± 6
Potassium (K^+^) (mg L^–1^)	4621 ± 9
Magnesium (Mg^2+^) (mg L^–1^)	2864 ± 11
Calcium (Ca^2+^) (mg L^–1^)	574.5 ± 0.7
Bromide (Br^–^) (mg L^–1^)	165 ± 0.3
Fluoride (F^–^) (mg L^–1^)	< 50
Nitrate (NO_3_^–^) (mg L^–1^)	< 50
Nitrite (NO_2_^–^) (mg L^–1^)	< 50

*Sample was diluted 1:5 with distilled water for measurement. Fluoride, nitrate and nitrite concentrations were below the detection limit.

### 2.2 Cultivation of archaea

Cultivation within this study focused on the enrichment and isolation of haloarchaea as they were hypothesized as one of the major taxa within the brine and among the best adapted microorganisms to hypersaline conditions. For solid cultivation of haloarchaeal isolates, artificial sea water (ASW) media for halophiles J457 was used: 195.00 g L^–1^ NaCl, 35.00 g L^–1^ MgCl_2_ × 6 H_2_O, 50.00 g L^–1^ MgSO_4_ × 7 H_2_O, 5.00 g L^–1^ KCl, 0.25 g L^–1^ NaHCO_3_, 1.00 g L^–1^ NaNO_3_, 0.50 g L^–1^ CaCl_2_ × 2 H_2_O, 0.05 g L^–1^ KH_2_PO_4_, 0.03 g L^–1^ NH_4_Cl, 0.05 g L^–1^ yeast extract, and 1.00 g L^–1^ sodium pyruvate at pH 7.4. In addition, filtered (0.44 μm), autoclaved Lunenburg brine supplemented with 1% (w/v) yeast extract and 0.75% (w/v) casamino acids was used as a culture media. For agar plates, 15 g L^–1^ agar was added and the media was autoclaved for 20 min at 121 °C. To screen for culturable archaeal microorganisms, 200 μL of untreated brine was spread on agar plates and were incubated at 37 °C for one month until growth occurred. Colonies were taken and single colonies were re-streaked several times to gain pure cultures and maintained on ASW. Two representative isolates were chosen (*Haloarcula* sp. NS06 and *Halorubrum* sp. AS12) to perform molecular and morphological analysis. For this, incubation temperature for *Haloarcula* sp. NS06 was increased to 42 °C to maintain the optimum growth conditions (Oren et al., 1990; Schwarzer et al., 2021), unless otherwise described.

In addition, liquid cultivation was performed using five different media, which were evaluated for their ability to enrich haloarchaeal communities: artificial seawater (ASW), Marine Broth (Difco) adjusted to 195 g L^–1^ NaCl, R2A medium (HiMedia) at 3.12 g L^–1^ prepared in 100% brine, a 1:1 (v/v) mixture of brine and R2A (1.56 g L^–1^), and brine supplemented with 0.1% (w/v) yeast extract and 0.075% (w/v) casamino acids. Each medium was inoculated with 5% (v/v) Lunenburg brine and incubated at 37 °C with shaking at 150 rpm. After 32 days, 2 mL of each well-grown culture were harvested by centrifugation (10 min, 14.100 g) and processed for amplicon sequencing as described in section “2.3 Molecular and metabolic analysis.” All enrichments were performed in triplicates.

### 2.3 Molecular and metabolic analysis

#### 2.3.1 Amplicon sequencing (V1–V4 regions)

Microbial diversity of enrichments as well as environmental DNA of Lunenburg brine were further assessed via 16S rRNA amplicon sequencing.

For environmental 16S rRNA gene sequencing, five replicates of 15 liters, amounting to 75 L of brine in total, were filtered through membrane filters (Merck, cellulose mixed esters, hydrophilic, pore size 0.45 μm), with an EZ-Fit Manifold and EZ-Stream Pump (Merck) in October 2023 directly at the German Salt Museum. Filters were stored aseptically on ice for transportation and at −20 °C in the lab until further extraction. The filters were transferred into 2 mL bead-beating tubes (0.1 and 0.5 mm, one filter per tube), followed by the addition of 750 μL of lysis buffer (ZymoBIOMICS). Mechanical disruption was carried out using a TissueLyser II (Qiagen) at 30 Hz for 10 min. After this step, cell lysis and subsequent DNA extraction were performed using the ZymoBIOMICS DNA Microprep Kit. To maximize DNA yield, slight modifications were introduced: an additional washing step with 500 μL of lysis buffer was performed to recover all cells from the filters, along with the collection and use of the total eluate throughout the extraction process. In the final step, DNA was eluted using 20 μL of nuclease-free water, prewarmed to 65 °C. The eluate was stored at −20 °C until further use. DNA concentrations of the five replicates revealed 6.6–17.05 ng L^–1^ of filtered brine (9.99 ± 4.33 ng L^–1^ filtered brine, *n* = 5, measured with a Qubit fluorometer), while no DNA could be quantified from an empty filter used as a negative control. The authors are aware that DNA extraction of *Halococcus* is beneficial using a phenol-dependent XS-buffer method (Leuko et al., 2008). However, extraction with the Microprep Kit (ZymoBIOMICS) yielded more DNA required for further sequencing, and also led to the identification of *Halococcus* representatives.

For analysis of enriched cultures, centrifuged pellets were resuspended in 300 μL distilled water and 450 μL lysis buffer and lysed with bashing-bead tubes. Further processing was conducted using the ZymoBIOMICS 96 MagBead DNA Kit with an Eppendorf DNA robot (epMotion 5075t). DNA yields ranged between 6.35 and 81.8 ng/μL^−1^ (as measured by TapeStation).

To elucidate the microbial diversity, extracted DNA from the brine and enrichments were analyzed by employing the Quick-16S NGS Library Prep Kit from ZymoBIOMICS (D6400) and the protocol for low biomass samples. Environmental DNA was analyzed via two separate sequencing runs involving the V1/V2 region of the 16S rRNA gene, used for the detection of bacteria and the V3/V4 region, amplifying a broad coverage of both bacteria and archaea. Enrichments were sequenced targeting the V3/V4 region only. In total, 2 μL DNA were used for the primary amplification steps consisting of 40 cycles. PCR conditions were adjusted according to the manufacturer’s instructions. In total, 10–12 pM DNA were loaded on the MiSeq for V1/V2 and V3/V4 sequencing, respectively. The ZymoBIOMICS Microbial Community DNA Standard from ZymoBIOMICS (Kit D6400) served as a positive control. The empty filter and a non-template control (NTC) served as negative controls and yielded negative results during the amplification process.

Sequencing results were further processed in R (Version 4.4.1) using FastQC (V0.12.0) (Andrews, 2010), the DADA2 package (V1.32.0) (Callahan et al., 2016) and visualized using VisuaR (Ruff Hrabe and de Angelis, 2023). First, the quality of the run was evaluated with the FastQC analyzer tool. Primers were trimmed on the left, according to the recommendation of the NGS Library Prep Kit (V1/V2): trimLeft < - c (19,16) and V3/V4: trimLeft < - c (16, 24). Reads were trimmed according to the quality profile determined by DADA2 to 270, 230 bp (V3, V4) and 230, 220 bp (V1, V2), filterAndTrim [fnFs, filtFs, fnRs, filtRs, maxN = 0, truncLen = c (270/230, 230/220)], trimLeft = trimLeft, maxEE = c (2, 2), truncQ = 2, rm.phix = TRUE, compress = TRUE, multithread = FALSE). For bacterial and archaeal taxonomic assignment, the database SILVA_nr99_V138.1 was utilized. Assigned taxonomic names can differ from current “List of Prokaryotic names with Standing in Nomenclature” (LPSN) usage, due to frequent updates to the database.

#### 2.3.2 16 S rRNA sequencing of axenic cultures

In total, 24 archaeal cultures were isolated and cultured in the laboratory. For the identification of the pure cultures, the 16S rRNA was targeted. Amplification of the 16S rRNA gene was achieved by performing colony PCR. For this, several colonies of a single isolate were diluted in 0.2 mL distilled water. A total of 1 μL of the sample was taken and mixed with 10 μL Luna^®^ Universal qPCR Master Mix (NEB) as well as 0.5 μL of 10 μM of each of the primers Halo 5F (5′ATTCGGTTGATCCTGCCGGA′3) and Halo1462R (5′CAGATTCCCCTACGGCTACCTT′3) (Leuko and Rettberg, 2017). The PCR reaction was filled up to 20 μL with 8 μL nuclease free water. For amplification, initial heating of the lid was performed at 95 °C for 15 min and 35 cycles, consisting of a denaturation step at 95 °C for 30 s, an annealing step at 55 °C for 30 s, and an elongation step at 72 °C for 90 s. Loop was closed with a final heating step at 72 °C for 10 min. Successful amplification was verified with an additional 1% agarose gel. DNA concentrations were measured using the Qubit fluorometer. Samples were further purified with the DNA PCR and Purification Kit (Monarch) and sequenced with the commercial Sanger Sequencing Service of Eurofins using the forward primer Halo 5F. For full-length analysis, samples were also sequenced with the reverse Halo1461R primer. Full-length 16S rRNA was assembled with MEGA 11 (Version 11.0.13) (Tamura et al., 2021).

#### 2.3.3 Whole-genome sequencing and analysis

Two representative isolates were chosen, *Haloarcula* sp. NS06 and *Halorubrum* sp. AS12, to perform whole genome DNA extraction and sequencing. Isolates were selected based on the predominant abundance of the two genera in the obtained cultures and isolates, particularly *Haloarcula* and *Halorubrum* as well as due to different morphology. The DNA of the isolates was acquired by using genomic tips (20 μg) from Qiagen and following its protocol. For this, archaeal isolates were grown in liquid ASW media for five (NS06, 42 °C) to seven days (AS12, 37 °C). The sample was concentrated to a final OD_600nm_ of 1–1.2. Sequencing was performed using Nanopore Oxford technology with the SQK-LSK114DE kit and a R10.4.1 Flow Cell with a sequence duration of 24 h. A final concentration of 500 ng DNA was used for the analysis. Due to increased genome size of NS06, this isolate was sequenced twice. Integrity was assured with TapeStation and quantity was measured using the Qubit fluorometer. Base calling was performed with the high-accuracy model. The genome assembly was performed using the Flye software (Kolmogorov et al., 2019) (settings -nano-hq, policing iterations = 3, minimum overlap = 3,000) on the web-based analysis platform Galaxy (Version 24.1.2) (Afgan et al., 2018). Annotation of the assembled genomes was carried out using the Prokka software (Version 1.14.6) (Seemann, 2014) through Galaxy. Quality of the assembled genomes involving completeness and contamination ratio were determined using the CheckM tool (Parks et al., 2015). Annotations of genes involved in salt adaptation, pigmentation, and polyhydroxyalkanoate (PHA) production were performed with Prokka v1.14.6 and supplemented by NCBI RefSeq annotations (GCF_051122765.1 for AS12 and GCF_051122755.1 for NS06, available at NCBI). These complementary approaches yielded divergent protein sets due to differences in intrinsic ORF prediction and in database composition and curation. Proteome-wide isoelectric points (pI) were calculated using the iep tool implemented in Galaxy (v5.0.0.1), which is based on EMBOSS (Rice et al., 2000) and implemented in Perl.

#### 2.3.4 Compatible solute analysis

For compatible solute analysis, samples were grown in ASW medium (with 20% NaCl and 0.5% glucose) after being inoculated from a preculture of the same medium. Three aliquots of 5 mL were harvested in the mid-exponential phase at an OD_600_ of 2.9 (NS06) and 0.3 (AS12), pelleted (5 min, 8,000 g) and stored without the supernatant at −80 °C until further analysis. The cell number at the timepoint of sampling was counted using a Neubauer counting chamber (*Haloarcula* sp.: 1.33 × 10^8^ cells per mL and *Halorubrum* sp.: 1.27 × 10^7^ cells per mL).

Pelleted cells were resuspended in 100 μL of acetonitrile/water (9:1, v/v) containing an aqueous internal standard mixture [D_3_-ectoine, D_6_-dimethylsulfoniopropionate (DMSP) and D_3_-gonyol, 500 nM final], then vortexed for 30 s as previously described (Azizah and Pohnert, 2022). Ultrasonic lysis was performed (6 cycles of 10 s pulses at 40% intensity) with a Bandelin Sonoplus ultrasound homogenizer. After centrifugation for 10 min at 16,100 g, 5 μL of the resulting supernatants were directly subjected to UHPLC/HRMS for analysis. Analysis was conducted on a Dionex Ultimate 3,000 system (Thermo Scientific) coupled to a Q-Exactive Plus Orbitrap mass spectrometer (Thermo Scientific). The system was equipped with a SeQuant ZIC-HILIC column (2.1 × 150 mm, 5 μm) and a SeQuant ZIC-HILIC guard column (2.1 × 20 mm, 5 μm) (Merck). Xcalibur was used for MS data processing. Electrospray ionization in positive mode was applied under the following conditions: temperature, 380 °C; spray voltage, 3,000 V; sheath gas flow, 60 arbitrary units; and aux gas flow, 20 arbitrary units (Azizah and Pohnert, 2022).

The analysis of zwitterionic metabolites via UHPLC was performed as previously described (Azizah and Pohnert, 2022; Azizah and Pohnert, 2024). Mobile phases were high-purity water LC-MS grade (Th Geyer GmbH) supplemented with 2% acetonitrile LC-MS grade (Th Geyer GmbH) and 0.1% formic acid LC-MS grade (Thermo Scientific) as solvent A and 90% acetonitrile with 10% of a 1 mM aqueous ammonium acetate solution LC-MS grade (LGC Promochem) as solvent B. The gradient program (10 min) was as follows: 1 min 100% at solvent B (isocratic), 5.5 min linear gradient from 100% to 20% solvent B, 0.6 min linear gradient from 20% to 100% solvent B, and 2.9 min equilibration at 100% solvent B (isocratic). The column was maintained at 25 °C, the flow rate was 0.6 mL min^–1^, and the injection volume was 5 μL. Data were acquired in full scan mode (*m/z* 75–200) at a resolution of 70,000. Before analyzing samples, blanks were repeatedly run to condition the UHPLC.

Commercial and synthetic standards were used for the identification of zwitterionic metabolites in the samples by comparison of MS and MS/MS spectra. Commercially available standards used were glycine-betaine (Sigma-Aldrich), proline (Sigma-Aldrich), trigonelline (Sigma-Aldrich), choline (Sigma-Aldrich), sarcosine (ABCR GmbH), 5-hydroxyectoine (Sigma-Aldrich), and ectoine (Sigma-Aldrich). Gonyol and DMSP were obtained by synthesis as described in previous studies (Gebser and Pohnert, 2013). Quantification was performed using a standard calibration curve, evaluating analyte peak areas relative to those of the internal standard. D_3_-ectoine was used as an internal standard for the quantification of ectoine and 5-hydroxyectoine. D_6_-DMSP was used as an internal standard for DMSP. For all other zwitterionic metabolites, D_3_-gonyol was used as the internal standard. D_3_-gonyol, D_6_-DMSP and D_3_-ectoine were synthesized in our laboratory based on published procedures (Gebser and Pohnert, 2013; Fenizia et al., 2020). For glycine-betaine, the calibration curve (*n* = 3) for the area of the molecular ion was y = 9.47 × 10^–3^ *x* with *r* = 0.9959, limit of detection (LOD) = 39.45 nM, limit of quantification (LOQ) = 135.91 nM; for gonyol y = 4.95 × 10^–4^ *x* with *r* = 0.9977, LOD = 28.01 nM, LOQ = 97.34 nM; for ectoine, y = 5.66 × 10^–3^ *x* with *r* = 0.9986, LOD = 20.77 nM, LOQ = 73.32 nM; for 5-hydroxyectoine, y = 1.19 × 10^–3^ *x* with r = 0.9975, LOD = 24.03 nM, LOQ = 83.30 nM; for proline, y = 3.77 × 10^–3^ *x* with *r* = 0.9962, LOD = 17.67 nM, LOQ = 62.57 nM; for choline, y = 5.14 × 10^–3^ *x* with *r* = 0.9967, LOD = 26.50 nM, LOQ = 92.65 nM; for sarcosine y = 1.24 × 10^–3^ *x* with *r* = 0.9960, LOD = 24.24 nM, LOQ = 85.16 nM; for trigonelline y = 1.00 × 10^–2^ *x* with *r* = 0.9967, LOD = 10.99 nM, LOQ = 39.18 nM; for DMSP y = 1.45 × 10^–3^ x with *r* = 0.9998, LOD = 8.08 nM, LOQ = 29.04 nM. All performed calibration curves passed Mandel’s test.

### 2.4 Morphological characterization of isolates

#### 2.4.1 Fluorescence and electron microscopy

For fluorescence microscopy of *Haloarcula* sp. NS06 and *Halorubrum* sp. AS12, cultures were inoculated with 1% (v/v) of a preculture and incubated for 10 days at 37 °C (AS12) and 42 °C (NS06). A total of 2 mL of the culture was washed with 2 mL of TN buffer (20% NaCl, 0.1 M Tris, pH 7.4) and resuspended in 200 μL of TN buffer. For PHA staining, 4 μL of Nile Red (80 μg mL^–1^ in DSMO) were added to 100 mL of cell suspension as previously described by Zuriani et al. (2013) for a final concentration of 3.1 μg mL^–1^. Cells were incubated at room temperature for 30 min and then washed twice with TN buffer and were resuspended in TN buffer. To visualize all cells, they were stained with DAPI. To overcome insufficient cell staining, the optimized DAPI staining protocol by Küper et al. (2010) was used. For this, 100 μL of staining solution containing 30 μL 2 M Na-acetate (pH 4.7), 50 μL 100 mM Na_2_-EDTA, 10 μL DAPI (0.2 mg mL^–1^), and 10 μL 1% SDS solution, was prepared. A total of 1 μL of DAPI staining solution was added to 8 μL of cell suspension. To decrease motility and fix the cells, one drop of fluorescent mounting medium (Dako, Sigma-Aldrich) was added. Fluorescence microscopy (Axio Imager M2, Zeiss) was performed using two channels: DAPI fluorescence was detected using a 395 nm beam splitter with excitation at 335–383 nm and emission at 420–470 nm, while Nile Red fluorescence was captured using a 605 nm beam splitter with excitation at 574–599 nm and emission at 612–682 nm.

For scanning electron microscopy of cells, 100 μL from a frozen (−80 °C) glycerol stock were transferred to 500 μL ASW media in a 2 mL Eppendorf tube and incubated for 14 days at 40 °C without shaking. From the sediment of this culture, 100 μL was subsequently plated on an ASW agar plate and incubated for 40 days at 40 °C. For scanning electron microscopy imaging, three agar coupons with colonies on top were excised with a 6 mm biopsy punch and fixed for 24 h in a 50 mM HEPES buffer solution containing 1% paraformaldehyde and 2.5% glutaraldehyde. Washing of cells were performed in 50 mM HEPES buffer and dehydration in ethanol (30%, 50%, 70%, 90%, 95%, 100%). Afterwards, all samples were dried overnight in hexamethyldisilazane and mounted on aluminum stubs. Additionally, they were sputter coated with an 8 nm layer of gold-palladium. Finally, samples were visualized in the SEM (ZEISS 1530 Gemini, Carl Zeiss Microscopy), operating at 3 kV with the in-lens secondary electron detector.

For transmission electron microscopy of ultrathin sections through cells, archaeal cells of ASW cultures were either filtrated on a polyester filter or sedimented by slow-speed centrifugation (100 g, 5 min). Cells on filter were high-pressure frozen as described by Schaudinn et al. (2019). Cells from sediments were introduced into hollow cellulose tubes (McDonald et al., 2010) and high pressure-frozen using omega platelets (Article No. 403/404, Engineering Office M. Wohlwend) and a respective holder tip (Article No. 402, Engineering Office M. Wohlwend). Freeze-substitution of all samples was performed in the automated freeze-substitution machine (AFS, Leica Microsystems) using 1% glutaraldehyde with 5% water in acetone. The substitution mixture was exchanged against pure acetone at 0 °C, and then, the samples were brought to room temperature for sequential infiltration with increasing concentrations of epon resin in acetone. After infiltration with epon/acetone (3 plus 1 mixture) over night, filter or hollow tubes with cells were embedded in pure resin which was polymerized at 60 °C for 2 days. Polymerized resin blocks were sectioned with an ultramicrotome (UC7, Leica Microsystems) at 60–70 nm section thickness and collected on filmed electron microscopy grids. Sections were post-stained with uranyl acetate and lead citrate and inspected with a transmission electron microscope (Tecnai Spirit, ThermoFisher), which operated at 120 kV. Images were acquired using a side-mounted CMOS camera (Phurona, EMSIS) with a resolution of 4,112 × 3,008 pixels. All cultures for electron microscopy were grown at 40 °C, individual cultivation times for selected images are mentioned in the figure caption. The light microscopy image Figure 2A was taken from a liquid culture of NS06 after incubation at 42 °C for two days using oil immersion at 100× magnification.

#### 2.4.2 Raman spectroscopy

For further pigment analysis, Raman spectra of single colonies of *Haloarcula* sp. NS06 and *Halorubrum* sp. AS12 were analyzed based on Bernatova et al. (2013), Mlynarikova et al. (2015), Siems et al. (2023). For this, isolates were cultured on ASW medium and incubated at 37 °C for 7 days. The commercial Renishaw Raman spectrometer (Renishaw inVia Raman Spectrometer, Renishaw plc., Wotton-underEdge, United Kingdom) was used to acquire Raman spectra from microbial colonies. In the course of the experiment, a 785 nm laser was used, which was provided and operational within the experimental setup. The diameter of the circular laser beam was approximately 2 μm, and the laser power after passing through the microscope lens was approximately 100 mW in the sample plane. A microscope objective was used to focus a laser beam onto a single colony (Leica, Wetzlar, Germany: N PLAN EPI, magnification 50×, numerical aperture 0.75, working distance 0.5 mm). Different parts of a colony were used for individual 15 s spectral acquisitions in the range 614–1,724 cm^–1^. At least three different colonies were considered for in total 10 measurements per isolate. Afterwards, the spectra were analyzed with an in-house standard multivariate principle component program implemented in MATLAB (MathWorks, Natick, MA, United States). To suppress any background fluorescence, all Raman spectra were treated with rolling circle filtering (10 passes, 700 points circle radius). High-frequency noise was removed using Savitzky-Golay filtering (2nd order, width 7 points) (Rebrošová et al., 2017; Barton et al., 2018). Then, normalization was performed (to the peak assigned to an amino acid phenylalanine at approx. 1,001 cm^–1^) (Bernatova et al., 2013; Mlynarikova et al., 2015; Siems et al., 2023).

## 3 Results

### 3.1 Hydrochemical characterization

The brine consists primarily of sodium chloride (302.25 g L^–1^), with sources of carnalite, gypsum, epsom salt, soda as well as iron oxide (Deutsches Salzmuseum [German Salt Museum], n.d.; Table 1). Further measurements from the sampling campaign in April 2024 revealed a pH of 6.4, a temperature of 15.5 °C and a salinity of 300.5 ppt (see Table 2). In addition, 2.98 mg L^–1^ oxygen, as well as 3.04–3.437 mg L^–1^ DOC content were measured. Also, salt ions and possible electron acceptors like SO_4_^2–^, NO_3_^–^ and NO_2_^–^ were investigated. Most prominent ions were Cl^–^ (177 g L^–1^) and Na^+^ (107 g L^–1^). Besides, SO_4_^2–^, K^+^, Mg^2+^, Ca^2+^ and Br^–^ ions were also detected in measurable concentrations. Concentrations of F^–^, NO_3_^–^ and NO_2_^–^ were below the detection limit of 50 mg L^–1^.

### 3.2 Cultivable archaeal diversity

In total, 24 archaeal isolates were cultivated from solid cultivation and identified as isolates from the genera *Halorubrum, Haloarcula*, *Halolamina*, and *Natrinema*, with 16S rRNA sequence similarities ranging from 98.4% to 99.7% according to the NCBI database (see Supplementary Table 1). Out of the 24 isolates, *Haloarcula sp*. NS06 and *Halorubrum* sp. AS12 were chosen for further analysis due to their high abundance in cultivation experiments. According to 16S rRNA sequencing, NS06 is related to *Haloarcula japonica* JCM 7785^T^ with a sequence similarity of 98.60%, thus falling below the species threshold of 98.7% (Chun et al., 2018), while also displaying a divergence in its 16S rRNA gene, because of its different copies (Cui et al., 2009). AS12 revealed the greatest similarity with *Halorubrum terrestre* strain JCM 10247^T^, with a sequence similarity of 99.63%, determined by Sanger sequencing, thus exceeding the proposed species threshold (Chun et al., 2018). Due to the divergence within the 16S rRNA sequence, cultures were further analyzed within genomic sequencing (see section “3.3.2 Whole genome sequencing and osmotic adaptions of *Halorubrum*. sp. AS12 and 466 *Haloarcula*. sp. NS06”).

Liquid enrichments of microorganisms from the Lunenburg brine were performed using different media and primarily lead to the enrichment of the archaeal genera *Haloarcula* and *Halorubrum*. Other genera, including *Halofilum*, *Halococcus*, *Aliifodinibus*, *Halospina*, *Halobacteria*, *Salicola*, *Halolamina*, *Halobacterium*, and *Haloterrigena* (related to the genus *Natrinema*), were also enriched, but to a lesser extent (see Supplementary Figure 1). Notably, *Halofilum* showed its highest abundance in enriched cultures with Marine Broth, whereas all other tested saline media compositions yielded maximal enrichment of the genera *Halorubrum* and *Haloarcula*.

### 3.3 Molecular and metabolic analysis

#### 3.3.1 Bacterial and archaeal diversity

**FIGURE 1:**
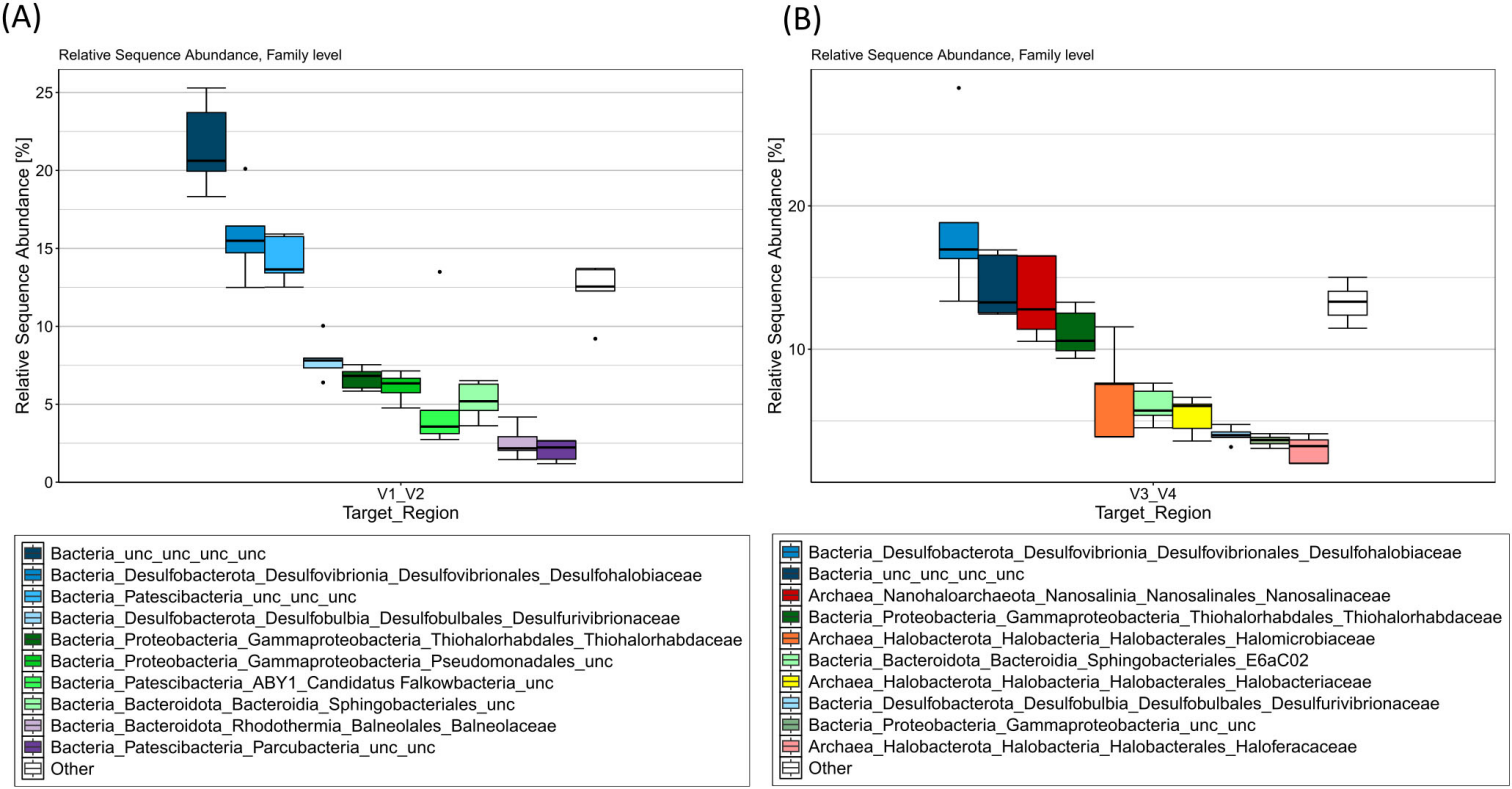
V1/V2 **(A)** and V3/V4 **(B)** 16S rRNA amplicon profiles from environmental DNA extracted from filtered brine water sampled at the Lunenburg brine (Germany). Taxa bar plots show the top 10 most abundant ASVs at the family level by relative sequence abundance (%). *n* = 5 samples (15 L each; 75 L total). Cool colors denote bacteria, warm colors denote archaea.

DNA extraction yielded in 6.6–17.05 ng L^–1^ brine (9.99 ± 4.33 ng L^–1^ brine, *n* = 5). No DNA could be quantified from an empty filter serving as a negative control. In total 73,450 and 505,538 reads could be determined after V1/V2 and V3/V4 sequencing, respectively. After quality trimming, 87.62% (V1/V2) and 92.06% (V3/V4) of the reads were maintained for further analysis. Following denoising, merging, and removal of chimeric sequences, 75.93 (V1/V2) and 67.87% (V3/V4) of the total reads were used for further taxonomic assignment. In total, 744 and 1,567 different amplicon sequence variants (ASVs) with 55,772 and 343,095 total reads were determined for V1/V2 and V3/V4 analysis, respectively. Within the V1/V2 analysis, 5.38% of ASVs were assigned to the kingdom of archaea, whereas for the V3/V4 analysis, 32.23% of ASVs were accounted to archaeal kingdom. Further sequencing data details on the analysis are displayed in the Supplementary Table 2. Figure 1 represents the 10 most abundant families determined by V1/V2 and V3/V4 sequencing. Corresponding relative sequence abundancies among phyla and family level are displayed in Supplementary Table 3.

The V1/V2 amplicon data revealed a clear dominance of the phyla *Desulfobacterota* (24.82%), *Patescibacteria* (24.26%), an uncultivated bacterial phylum (21.80%), *Proteobacteria* (14.20%) and *Bacteroidota* (9.67%). Minor taxa, within the top 10 abundant phyla detected, include the phyla *Halobacterota*, *Nanohaloarchaeota*, *Halanaerobiaeota*, *Verrucomicrobiota*, and *Chloroflexi* (see Supplementary Table 3). At the family level (see Figure 1A), an uncultivated bacterial family (21.80%), *Desulfohalobiaceae* (15.65%), as well as an uncultivated family among the *Patescibacteria* (14.34%) were sequenced in high abundance, suggesting their importance within this habitat.

Among the V3/V4 analysis, the phyla *Desulfobacterota* (22.87%), *Halobacterota* (15.73%), *Proteobacteria* (15.08%), an uncultivated bacterial phylum (14.51%), *Nanohaloarchaeota* (13.83%), and *Bacteroidota* (7.43%) were detected as the most represented and likely reflect key adaptations to the extreme environmental conditions. Among the top 10 most frequent phyla, minor abundances were detected for the phyla *Patescibacteria* (3.14%), *Aenigmarchaeota* (2.23%), *Nanoarchaeota* (1.13%), and *Chloroflexi* (0.81%). Within the most prominent bacterial families (Figure 1B), *Desulfohalobiaceae* (18.15%), an uncultivated bacterial family (14.51%), *Thiohalorhabdaceae* (11.14%), a family among the *Sphingobacteriales*_E6aC02 order (6.15%), *Desulfovibrionaceae* (4.04%), and an uncultivated family from the gammaproteobacterial class (3.59%) were detected as the most frequent taxa. Among the most common archaeal families, *Nanosalinaceae* (13.83%), as well as three families from the *Halobacteriales* order: *Halomicrobiaceae* (6.90%), *Halobacteriaceae* (5.39%), and *Haloferaceaea* (2.99%) were detected. The extremely halophile bacterial genus *Salinibacter* was detected within 163 (V1/V2) to 752 reads (V3/V4), thus accounting for 0.29% and 0.22% of the total reads.

One of the most frequent sequenced ASV within the V1/V2 and V3/V4 analysis (13.93% and 14.29% of the total ASV counts) revealed to be an unassigned and uncultured bacterium, only assigned to the domain level (see Supplementary Table 4). Further blasting among the NCBI database revealed its slight similarity (83.39%–93.75%) within an organism isolated from the Orca Basin, the largest hypersaline seafloor brine basin in the Gulf of Mexico (Nigro et al., 2016), which can be further assigned to *Patescibacteria* within SILVA’s taxonomy server. In fact, this ASV was just one single nucleoid different to the first most abundant ASV (14.00%) among the V1/V2 analysis which was already assigned to the *Patescibacteria* phylum.

#### 3.3.2 Whole genome sequencing and osmotic adaptions of *Halorubrum.* sp. AS12 and *Haloarcula.* sp. NS06

Most sequenced archaeal phyla within the V3/V4 sequencing was *Halobacterota* (15.73%), which also accounts the cultivated and enriched families *Halorubrum* and *Haloarcula*, which constitute 0.34 to 0.89% relative abundance of all generated reads. Whole genome sequencing (WGS) of the isolates *Halorubrum* sp. AS12 and *Haloarcula* sp. NS06 were performed to further elucidate the isolates genetic characteristics and adaptions to the Lunenburg brine, as well as their correct taxonomic classification. Estimated genome size of *Halorubrum* sp. AS12 and *Haloarcula* sp. NS06 were 3,255,470 and 4,579,968 bp and a GC content of 66.83% and 60.20%, respectively. Quality assessment revealed a completeness of 94.79%–98.85% and contamination ratio of 0%–1.6% for AS12 and NS06, respectively (see Supplementary Table 5). Prokka annotations are displayed in Supplementary Table 6 (*Haloarcula* sp. NS06 and Supplementary Table 7 (*Halorubrum* sp. AS12).

For *Halorubrum* sp. AS12, 3361 coding sequences (CDS) and 2152 hypothetical proteins (64%) were detected within annotation, whereas for *Haloarcula* sp. NS06, 4,919 coding sequences and 3,240 (66%) hypothetical proteins were discovered within the annotation using Prokka (see Supplementary Tables 5–7). The full circular genome could be assembled for AS12 displaying one primary chromosome (2,889,578 bp) including two copies of the 5S, 16S, and 23S rRNA operon, as well as one circular plasmid (365,892 bp) (see Supplementary Table 5). The two 16S rRNA genes, discovered through genomic annotation, lead to the same species identification as identified by Sanger sequencing, with a similarity of 99.12%–99.73% to *Halorubrum terrestre* JCM 10247^T^ (Supplementary Tables 1, 8). Further analysis of other highly conserved genes, which are only present once within the genome, revealed the closest similarity to *Halorubrum ruber* MBLA0099^T^ (*rpob”, secY, tuf, radA*) and *Halorubrum trapanicum* JCM 10477^T^ (*rpob*’) (Supplementary Table 8).

Genome assembly of *Haloarcula* sp. NS06 unveiled a more complex genomic structure, yielding 9 contigs. The primary circular chromosome (3,115,374 bp) harbors two copies of the rRNA operon, while a secondary circular chromosome (398,639 bp) contains a third 5S, 16S and 23S rRNA operon (Supplementary Table 5). In addition, the presence of a partial 5.8S rRNA (109 bp) was detected within all three RNA operons. In contrast to the 16S rRNA sequence, the partial 5.8S rRNA sequences were 100% identical in all RNA operons. A blast analysis of this gene on NCBI showed a 100% match to the genome sequences of *Haloarcula marismortui* ATCC 43409^T^ and *Haloarcula hispanica* N601^T^. Also, the assembly revealed four circular and one non-circular plasmid, while one of the replicons encodes an extra 5S rRNA copy and two plasmids harbor further tRNA.

Two 16S rRNA copies within the genome of NS06 revealed to be nearly identical (99%), both leading to the identification of *Haloarcula marismortui* ATCC 43049^T^ with sequencing similarities of 99.25 to 99.32%, while being located on different chromosomes. The third sequence was revealed to be different (95%) and has the highest similarity of 99.52% with *Haloarcula japonica* JCM 7785^T^. Further, highly conserved genes, only encoded on the primary chromosome, revealed closest similarity to *Haloarcula marismortui* ATCC 43049^T^ (*tuf, radA*’ and *radA*”), *Haloarcula argentinensis* JCM 9737^T^ (*rpob*’), *Haloarcula hispanica* ATCC 33960^T^ (*secY*) and *Haloarcula sinaaiensis* ATCC 33800^T^ *(rpob”)*. For further details see Supplementary Table 8.

To assess salt adaptation mechanisms, we screened both genomes for genes encoding light-driven ion pumps, K^+^, Na^+^ and Mg^2+^ transporters and compatible-solute pathways, using gene sets and functions compiled from previous studies (Schobert and Lanyi, 1982; Corratgé-Faillie et al., 2010; Resch et al., 2010, 2011; Youssef et al., 2013; Becker et al., 2014; Gunde-Cimerman et al., 2018; Straková et al., 2025). For this, annotations of the two isolates genomes performed by Prokka (Supplementary Tables 6, 7) and NCBI RefSeq assemblies (GCF_051122765.1 for AS12 and GCF_051122755.1 for NS06, available at NCBI) were used. Both strains contain the light-driven pump bacteriorhodopsin, however, halorhodopsin was detected only in *Haloarcula* sp. NS06 and is absent from *Halorubrum* sp. AS12 (see Supplementary Table 9). With respect to K^+^ homeostasis, both isolates harbor genes related to *NhaP2*, *NhaC*, *Kef*, *Trk*, *KdpABC* as well as mechanosensitive channels and further unspecified potassium channels. The additional channel *MthK* was found exclusively in *Haloarcula* sp. NS06. Overall, *Haloarcula* sp. NS06 carries a broader repertoire of K^+^ antiporters and pumps. For Mg^2+^ transport, *Haloarcula* sp. NS06 encodes for *CorA*, which allows active diffusion. Regarding compatible solutes, both strains possess *BetA* (choline dehydrogenase) for glycine-betaine synthesis. Genes for trehalose biosynthesis are present in both genomes, but *Halorubrum* sp. AS12 relies on trehalose glucosyltransferase (*TreT*), whereas *Haloarcula* sp. NS06 employs the trehalose-6-phosphate synthase/phosphatase (*TpsP*/*OtsAB*). *Haloarcula* sp. NS06 additionally encodes further genes related to the BCCT (betaine/carnitine/choline transporter) family and an extra gene involved in glycine-betaine transport. In contrast, *Halorubrum* sp. AS12 harbors genes (*ProA, ProB* and *ProC*) for proline synthesis from glutamate, which are absent in *Haloarcula* sp. NS06. No genes for ectoine biosynthesis were detected within the genomes of *Haloarcula* sp. NS06 and *Halorubrum* sp. AS12.

The isoelectric point (pI) distribution was highly similar in *Halorubrum* sp. AS12 and *Haloarcula* sp. NS06, despite NS06 encoding a larger number of sequences overall (Supplementary Table 6 and 7). The pIs of the proteome were calculated to be acidic, with average values of 4.92 for *Haloarcula* sp. NS06 and 4.87 for *Halorubrum* sp. AS12. Most proteins were acidic (pI ≤ 5; 81.2% in AS12 and 78.8% in NS06), including enzymes, ribosomal proteins, and the majority of DNA-processing enzymes. Extremely acidic proteins with a pI below 3 (4 in AS12 and 6 in NS06) were exclusively hypothetical in both isolates. Proteins with a pI below 4 included CDS essential for DNA replication and repair, such as the DNA double-strand break repair protein Mre11, the DNA-directed RNA polymerase subunits D and L, and the primase DnaG in both isolates. Coding sequences for polyhydroxyalkanoate (PHA) synthesis in NS06 also exhibited pI values below 4. A small fraction of alkaline proteins with a pI > 9 (6.87% in AS12 and 7.01% in NS06) were mainly hypothetical, but also included 30S and, more prominently, 50S ribosomal proteins as well as membrane-associated proteins.

The concentration of the tested intracellular compatible solutes measured by UHPLC was low (See Supplementary Table 10). For both isolates minor amounts of glycine-betaine, 5-hydroxyectoine, proline, ectoine, gonyol, sarcosine, trigonelline and choline could be detected. In general, concentrations were higher in *Halorubrum* sp. AS12 compared to *Haloarcula* sp. NS06. However, their overall concentration was below 0.1 fmol cell^–1^. Highest concentrations (above 0.001 fmol cell^–1^) were found for proline, glycine-betaine, ectoine, sarcosine and choline in *Halorubrum* sp. AS12 with a clear dominance of proline (0.02 fmol cell^–1^). In *Haloarcula* sp. NS06, glycine-betaine represented the most abundant solute, although at a comparably low level of 0.0007 fmol cell^−1^.

### 3.4 Morphological analysis of *Halorubrum*. sp. AS12 and *Haloarcula*. sp. NS06

#### 3.4.1 Cell morphology

**FIGURE 2:**
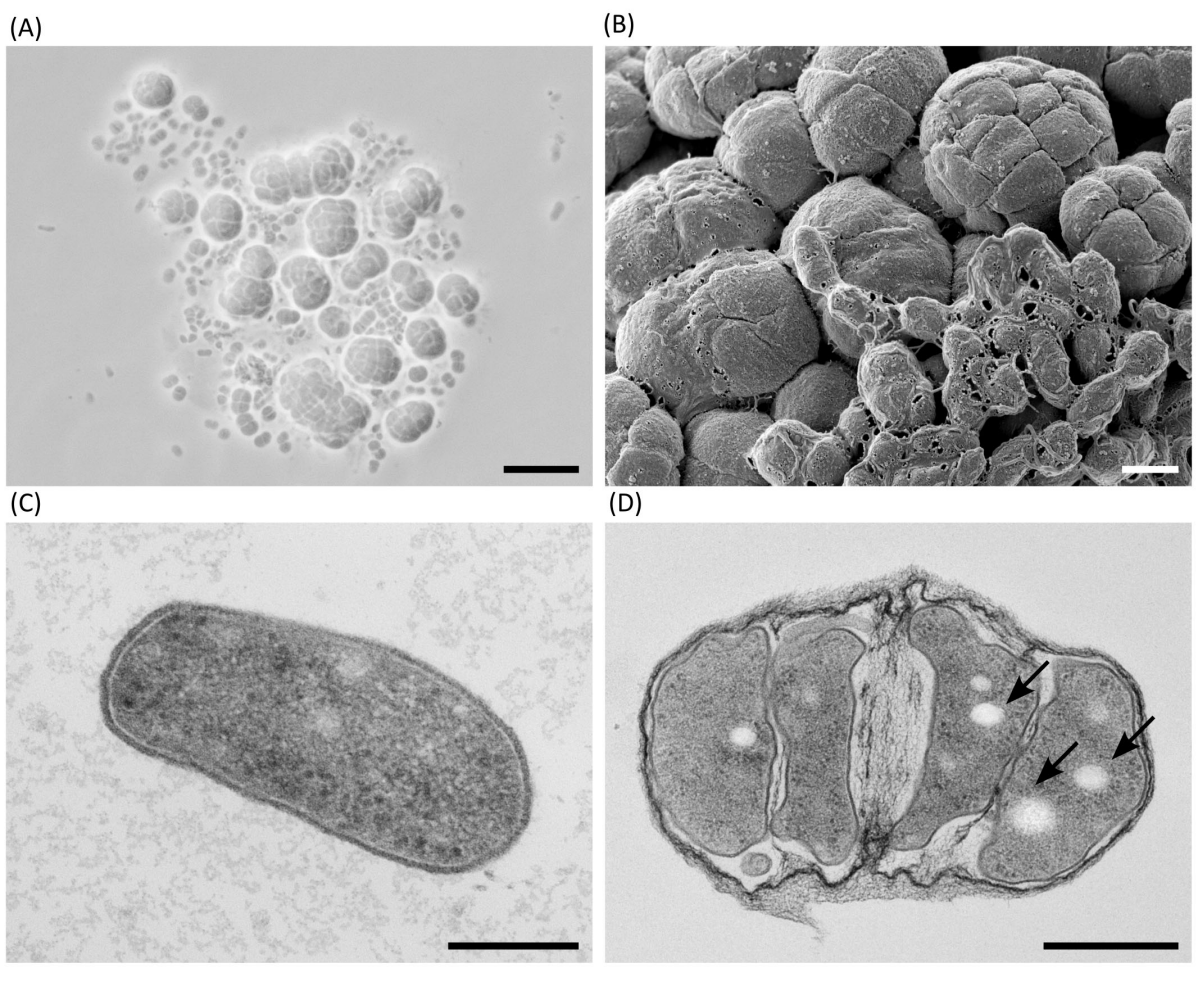
Microscopic morphology of *Haloarcula* sp. NS06 in light **(A)**, scanning electron **(B)** and thin section electron microscopy **(C,D)**. **(A)** In a 2-days-old artificial sea water (ASW) suspension culture, single cells are pleomorphic in shape, from round to rod-like, and frequently found in short chains or small aggregates. In addition to single cells and smaller groups, larger aggregates of larger cells are visible, which we have termed “package-like aggregates.” **(B)** Larger assemblies of package-like aggregates and short chains of single cells on top of them are visible, if cells are cultivated on agar for 3 weeks. **(C)** Transmission electron microscopy of a thin section through a single rod-shaped cell of a 23-days-old suspension culture shows a dense cytoplasm containing a few circular regions of lower density and some denser structures, which most likely represent ribosomes. The cell is limited by a thin and dense layer, which is separated from the plasma by a bright appearing gap most probably representing the central part of the plasma membrane. **(D)** Transmission electron microscopy of a thin section of a 23-days-old suspension culture through a larger group of cells which are enclosed by fibrous sheath. The bright-appearing inclusions (arrows) are visible in most section profiles. Panel **(B)** shows a composite micrograph generated by merging two images acquired at different focal planes. Scale bars represent 10 μm **(A)**, 1 μm **(B)**, 0.2 μm **(C)** and 0.5 μm **(D)**.

**FIGURE 3:**
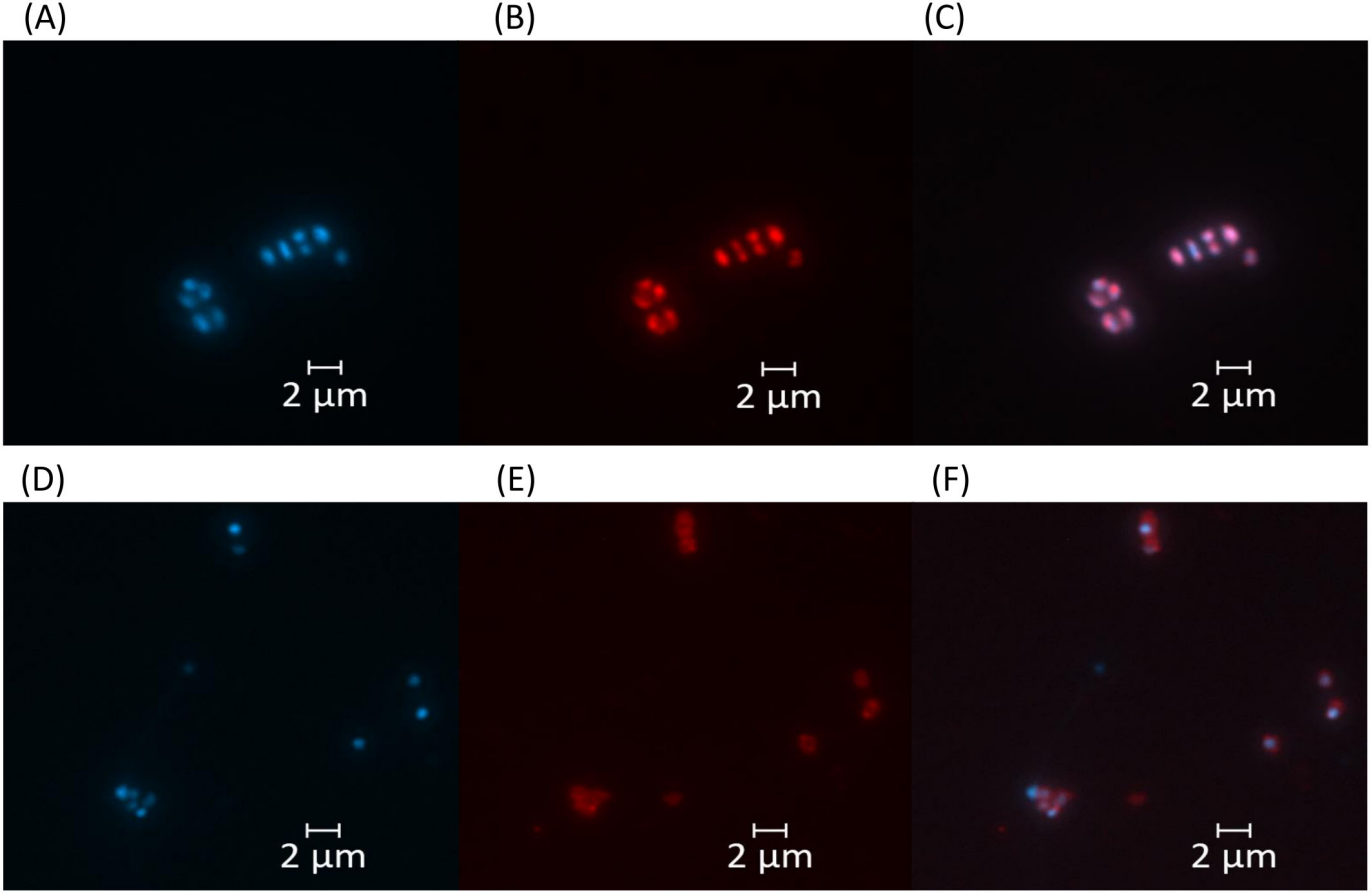
Fluorescence microscopy images of *Haloarcula* sp. NS06 **(A–C)** and *Halorubrum* sp. AS12 **(D–F)**. Left panels **(A,D)** show DAPI fluorescence channel for DNA visualization, middle panels **(B,E)** display Nile Red fluorescence channel for whole cell stain, and right panels **(C,F)** represent the merged images of both channels. Cell inclusions appeared red within fluorescence microscopy after staining with the lipophilic dye Nile Red **(B,E)**, although staining intensity was higher in *Haloarcula* sp. NS06 **(B)** than in *Halorubrum* sp. AS12 **(E)**. Scale bars represent 2 μm.

*Haloarcula* sp. NS06 cells in suspension cultures appeared as small and pleomorphic, spherical and rod-shaped, single cells or short chains, and, predominantly, as larger clusters of spherical units which we term “package-like aggregates” (Figure 2A). Cells cultivated on agar revealed larger assemblies of these package-like aggregates with single-celled forms at their surface (Figure 2B). In thin section electron microscopy, single cells reveal a largely dense cytoplasm with a few small circular regions of lower electron density (Figure 2C). Few dense particles of about 20 nm in width resemble ribosomes (Figure 2C). The cells are limited by a thin and dense layer which is followed by a bright-appearing thin space and the plasma membrane (Figure 2C). Larger aggregates of cells are formed by larger and more pleomorphic cells which are surrounded by a fibrous sheath (Figure 2D). The cytoplasm and wall structures appear similar to the corresponding structures in the single cells with the exception that the plasma regularly reveals several bright-appearing inclusions (Figure 2D). The inclusions could be formed by lipids, PHA or even gas. Inclusions appeared red within fluorescence microscopy after staining with the lipophilic dye Nile Red (Figure 3). Genomic analysis revealed the presence of genes involved in the PHA production within *Haloarcula* sp. NS06: Poly(3-hydroxyalkanoate) polymerase subunit *PhaE* and Poly(3-hydroxyalkanoate) polymerase subunit *PhaC* (Muangsuwan et al., 2015) (Supplementary Table 6).

Cells of *Halorubrum* sp. AS12 from suspension cultures appear slightly smaller and ovoid- to coccoid -shaped as cells of *Haloarcula* sp. NS06 (see Supplementary Figure 2A). Transmission-electron microscopy further revealed different morphologies including thin rods and coccoidal shaped cells within colonies (Supplementary Figure 2B). Less frequent and bright inclusions were also detected. Smaller aggregates of cells, which are connected by fine fibrous material were observed for *Halorubrum* sp. AS12, but no package-like aggregates (Supplementary Figure 2B). Cells within colonies are interconnected by a complex matrix (Supplementary Figures 2C, D). Cells of *Halorubrum* sp. AS12 contained lipophilic components, as shown by Nile Red fluorescence staining, although the staining intensity was lower than for *Haloarcula* sp. NS06 (Figure 3). However, genes involved in the PHA production, as indicated above, could not be detected for *Halorubrum* sp. AS12.

#### 3.4.2 Pink pigmentation

**FIGURE 4:**
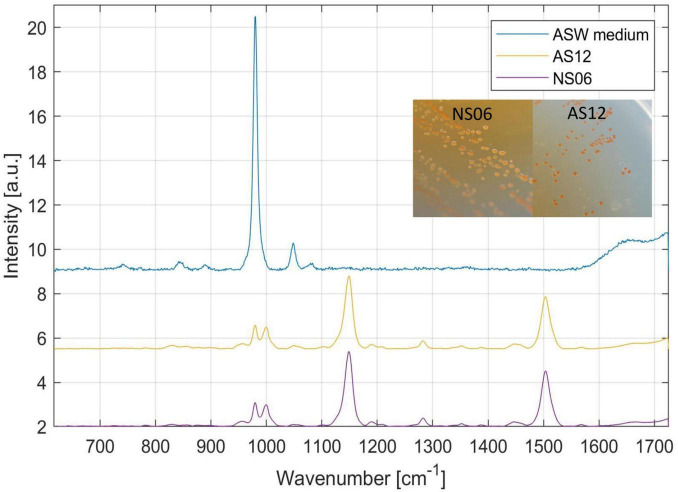
Raman spectroscopy and colony formation on artificial sea water (ASW) media of the two isolates *Haloarcula* sp. NS06 and *Halorubrum* sp. AS12 as well as the media as a reference. Raman spectra revealed main peaks for NS06 and AS12 at 980–1,000 (double peak), 1,147 and 1,504 cm^−1^, typical for carotenoids. In addition, minor peaks were recognized at 781, 828, 956, 1,048, 1,191, 1,282, 1,353, 1,389, 1,448, 1,567, and 1,664 cm^–1^. The first peak can be assigned to the internal background signal from the ASW media itself, since the media is showing a similar peak at 980 cm^−1^.

Colonies of the isolates *Halorubrum* sp. AS12 and *Haloarcula* sp. NS06 showed a distinct, pink pigmentation. However, AS12 showed more intensive pigmentation than NS06. Colonies of NS06 exhibit slightly irregular edges, while the colonies of AS12 have well-defined, smooth edges (see Figure 4).

To identify the pink pigments, colonies of *Haloarcula* sp. NS06 and *Halorubrum* sp. AS12 were further analyzed with Raman spectroscopy. Raman spectra revealed main peaks for NS06 and AS12 at 980–1,000 (double peak), 1,147 and 1,504 cm^−1^, typical for carotenoids. In addition, minor peaks were recognized at 781, 828, 956, 1,048, 1,191, 1,282, 1,353, 1,389, 1,448, 1,567 and 1,664 cm^–1^. The first peak can be assigned to the internal background signal from the ASW media itself, since the media is showing a similar peak at 980 cm^−1^. Within a different study, identical peaks were also detected for the carotenoid bacterioruberin within *Halobacterium salinarum* NRC-1, *Halococcus morrhuae* and *Natrinema pallidum* with three peaks at 1,505, 1,152, 1,000 cm^–1^ (Marshall et al., 2007).

Within both isolates, we found similar genes responsible for pigmentation including carotenoid 3,4-desaturase (*crtD*), lycopene elongase (*lye*), bisanhydrobacterioruberin (*cruF*) hydratase as well as two copies of beta-carotene 15,15’-dioxygenase(*brp*) and lycopene beta-cyclase (*crtY*) (Yang et al., 2015), encoded on the primary chromosome of both isolates. Identification of these genes support the detection of the pink bacterioruberin within both isolates.

The gene *bop* encoding the electron pump bacteriorhodopsin, which exhibits similar, yet more complex, Raman spectra (Marshall et al., 2007), was also identified in NS06, inserted between the genes coding for the B and D subunits of the V-type ATPases operon (see Supplementary Table 6). However, in AS12, the bacteriorhodopsin gene was not detected within the annotation via Prokka. Instead, four hypothetical proteins were found located between the genes coding for the B and D subunits of the V-type ATPase operon (Supplementary Table 7). Using the NCBI RefSeq/PGAP annotation, we additionally identified a bacteriorhodopsin gene in AS12 (see Supplementary Table 9) at a distinct locus, non-syntenic with the V-type ATPase operon (see NCBI RefSeq assembly GCF_051122765.1).

## 4 Discussion

This study presents the first comprehensive analysis of the microbial diversity of the hypersaline brine in Lunenburg, a halite remnant of the Zechstein Sea in northern Germany. By integrating 16S rRNA amplicon sequencing (V1/V2 and V3/V4) with additional cultivation, enrichment and characterization of isolated haloarchaea, it provides a first overview of the present microbial community. Two isolates, representing the most abundant cultivated genera were further examined by whole-genome sequencing, pigment profiling, cell morphology analysis (including screening for intracellular PHA), as well as osmoadaptation strategies.

### 4.1 Environmental interpretation of brine hydrochemistry on Earth and other planetary systems

For prediction of Earth-like life on other celestial bodies, the most important parameter is liquid water (Martin and McMinn, 2018). However, to remain liquid at subzero temperatures, water may persist as high-salinity brine, since high salt concentrations depress the freezing point of water and expand the habitability of cold environments (Mason and Elwood Madden, 2022; Cesur et al., 2022). In particular, perchlorate salts at elevated levels, such as 44.0% (w/v) magnesium perchlorate, can enable the persistence of liquid brines at temperatures as low as −67 °C (Chevrier et al., 2009). Thus, brines are promising targets in the search for life on extraterrestrial moons and planets (Mancinelli et al., 2004; Renno et al., 2021). In hypersaline ecosystems in space and on Earth, microorganisms have to cope with high osmotic pressure, low water activity and limited nutrient availability. Background radiation from halite-associated isotopes can also affect long-term survival (Kminek et al., 2003). The Lunenburg brine, characterized within this study, is representing a terrestrial analogous habitat to further test the limits of life and microbial diversity at hypersaline brines also found in space.

The brine characterized within this study has a NaCl concentration of 302.25 g L^–1^ (see Table 1), which is close to its saturation point. The high salinity results from groundwater leaching halite formations. While the major detected ions were Cl^–^ (177g L^–1^) and Na^+^ (107 g L^–1^), other osmorelevant ions such as Mg^2+^, K^+^ and SO_4_^2–^ were detected and contribute to increased osmotic pressure (see Table 2). Depending on the dominant salt type, water activity, which corresponds to water availability, in saline ecosystems can range from 0.982 (sea water) to 0.742 (NaCl saturated brine), and can go as low as 0.409 (MgCl_2_ dominated brine) (Paris et al., 2023), which clearly limits microbial life and survival. A study investigating the lower experimental minima of water activity necessary for growth revealed values of 0.803–0.642 for halophilic archaea and bacteria (Stevenson et al., 2015). High concentrations of NaCl and MgCl_2_ in brines increase the osmotic pressure on microorganisms, while major adaptation mechanisms within the Lunenburg brine have to account for the high sodium and chloride content. Identified adaptation mechanisms are further discussed and characterized via genome sequencing and compatible solute analysis (see section “4.3.2 RNA operons variation and osmotic adaptation within cultivated halophilic archaea”).

Next to Na^+^, Cl^–^, and Mg^2+^ (mainly in halite, carnallite and epsom salt), K^+^ also occurred in measurable amounts in Lunenburg, chiefly in carnallite and potassium chloride (see Tables 1, 2). The detected K^+^ amount of 4.6 g L^–1^ may arise radiation stress, accumulating over time. As mentioned earlier, radiation represents an additional stress factor in halites, due to natural background radiation from the isotope ^40^K, which accounts 0.0117% of natural potassium these days, and displays a relevant radioactive isotope with a similar concentration on Mars (Kminek et al., 2003). Based on Kminek et al. (2003), without any active DNA repair mechanism in the dormant phase, radiation limits the survival of even highly resistant bacterial endospores in halites on Earth to 109 million years. Hence, isolating of spores without active repair mechanisms from 250 million-year old halite fluid inclusions, which would have accumulated 28–88 kGy, for 2–7 g L^–1^ potassium, respectively, is likely impossible (Kminek et al., 2003).

The dissolved organic carbon (DOC) content is influenced by vegetation, including microbial and plant metabolism, and varies in aquatic systems from 1.8 mg L^–1^ in groundwater to 4.3 mg L^–1^ in river/streams to 21.3 mg L^–1^ in lakes, according to a study which analyzed 261 aquatic samples types (Ma et al., 2022). Research on DOC in North Pacific seawater reported even lower concentrations of 33–102 μM (equivalent to 0.40–1.23 mg L^–1^ assuming DOC is reported as C) with DOC decreasing from surface down to 1,000 m depth (Ge et al., 2022). In our study, DOC concentrations ranged from 3.04 to 3.427 mg L^–1^ indicating higher levels than in groundwater and seawater, but lower levels compared to rivers and streams (see Table 2). The elevated DOC values may result from the precipitation and concentration of dissolved organics derived from the ancient Zechstein Sea or from ongoing microbial production. Given the sampling depth and absence of light, phototrophic DOC production can be excluded.

The dissolved-oxygen concentration in the brine was low (2.98 mg L^–1^, Table 2) and thus slightly higher than in a recent study investigating non-saline groundwater with oxygen levels of 0.52 mg L^–1^ (Ruff et al., 2023). Low oxygen levels suggest an ecosystem dominated by facultative or obligate anaerobic microorganisms, as also indicated by amplicon sequencing with overall high abundancies of anaerobic *Desulfohalobiaceae* and *Desulfurivibrionaceae* as well as facultative anaerobic *Thiohalorhabdaceae* (Figure 1). Yet, electrochemical measurement of dissolved-oxygen in salt saturated samples is impaired by salinity and would require a 10-fold sample dilution. Measurable sulfate concentrations were detected (Table 2), consistent with the high relative abundance of anaerobic, sulfate-reducing *Desulfohalobiaceae* (Figure 1). Likewise, facultatively anaerobic *Thiohalorhabdaceae* were sequenced as abundant microorganisms within the brine, oxidizing reduced sulfur compounds for energy production using either nitrate or oxygen as electron acceptors (Sorokin and Merkel, 2025). However, neither nitrate nor nitrite were detected, suggesting that the use of these compounds as terminal electron acceptors is not a major metabolic pathway in this environment.

### 4.2 *Halorubrum* and *Haloarcula* as dominant cultivated genera in oxic cultivation

With oxic cultivation approaches, representatives from the *Halobacteriales* order including the genera *Haloarcula, Halorubrum, Halolamina*, and *Natrinema* were isolated (Supplementary Table 1). Cultivation and enrichment experiments were performed oxically, focussing on non-methanogenic, halophilic archaea which are mostly described as aerobic heterotrophs or facultative anaerobic. However, two exceptions, being obligate anaerobic haloarchaea, are the genera *Halanaeroarchaeum* (Sorokin et al., 2016) and *Halodesulfurarchaeum* (Sorokin et al., 2017). Some haloarchaea are able to perform light-driven generation of ATP via bacteriorhodopsin and halorhodopsin, while a low oxygen concentration even promotes production of bacteriorhodopsin within cells of *Halobacterium salinarum* (Oren, 2013). Some species like *Haloarcula marismortui* and *Haloferax volcanii* are also able to perform denitrification under anaerobic conditions, utilizing nitrate as the terminal electron acceptors. However, anaerobic growth revealed slower growth than in aerobic conditions for *Haloferax* strains (Hattori et al., 2016). With solid and liquid cultivation, the genera *Halorubrum* and *Haloarcula* were among the most abundant (Supplementary Table 1 and Supplementary Figure 1). This finding aligns with previous reports, highlighting these two genera as among the most frequently cultivated from hypersaline environments (Mani et al., 2012; Wen et al., 2018). Their corresponding families, *Haloferacaceae* and *Halomicrobiaceae*, were also among the most sequenced archaeal taxa in the environmental 16S rRNA amplicon dataset, together accounting for approximately 10% of the total V3/V4 reads (see Supplementary Table 3).

Marine Broth supplemented with NaCl primarily led to the enrichment of Halofilum (Supplementary Figure 1), a halotolerant bacterial genus, which tolerates salt levels from 2% to 20% (Xia et al., 2017). One possible explanation for this result is the lower concentrations of MgCl_2_ and KCl in the medium, which halophilic archaea require to maintain osmotic balance. Further information on genomic adaptations and the morphology of the isolates are discussed in se section “4.3 Molecular and metabolic analysis” and “4.4 Morphological variability.”

### 4.3 Molecular and metabolic analysis

#### 4.3.1 Microbial and molecular diversity within the Lunenburg brine

The amplicon data within the study predominantly identified the phyla *Desulfobacterota, Patescibacteria*, and *Halobacterota* as a characterizing community and complement previous research on related saline environments from ancient Zechstein Sea (Figure 1 and Supplementary Table 3). A study investigating the Boulby Mine in the United Kingdom, which also exploits the Zechstein evaporite deposits, used this habitat as an extraterrestrial analog for research (Cockell et al., 2020; Mathanlal et al., 2021). Analysis of the metagenome revealed, that the brines within the Boulby Mine were dominated by the archaeal order *Halobacteriales*, with *Haloarcula, Natronomonas, Halogeometricum, Halomicrobium*, and *Halobacterium* being the most sequenced genera (Payler et al., 2019). Even though a major part of the sequences within this study were assigned to the bacterial kingdom, we were also able to detect several families from the order *Halobacteriales* and *Nanosalinales*.

The yet-uncultivated and just recently proposed *Nanosalinaceaea* family (Rinke et al., 2021) was the most prominent archaeal family within this study’s sequencing results, deciphering a difference to the study on the Boulby Mine (Payler et al., 2019), and the analysis of the ancient Zechstein caves in Germany (Schwab et al., 2022; Figure 1B). Since their description, they were detected within soda-saline lakes in China (Zhao et al., 2022), acidic saline lakes in Australia (Boase et al., 2024) and a crystallizer pond in Spain (Liébana et al., 2024), but also a polish salt mine (Lach et al., 2023). Given the recent increase in metagenomic detections of *Nanohaloarchaeota*, it is plausible to hypothesize, that a reanalysis of the sequence data from the Boulby Mine study and caves in northern Germany, including other earlier studies investigating Permo-Triassic salt (Radax et al., 2001), using updated annotation methods and databases, might uncover the presence of *Nanohaloarchaeota*. The Candidate phylum *Nanohaloarchaeota*, which belongs to the DPANN superphylum (consisting of *Diapherotrites, Parvarchaeota, Aenigmarchaeota, Nanoarchaeota, Nanohaloarchaeota*), is characterized by its small cell size (Hamm et al., 2019). Members of this phylum were recently reported to be symbionts requiring a host from the *Halobacteria* class (Hamm et al., 2019; La Cono et al., 2020), and it was suggested that they may even have co-evolved together (Zhao et al., 2022).

Research investigating several salt caverns in mid Germany descending from the ancient Zechstein Sea detected also *Desulfobacterota*, next to halophilic archaea, as one of the core phyla within the ancient Zechstein formations (Schwab et al., 2022). Similar observations have been made in this study. In terms of the bacterial kingdom, we were able to detect *Desulfohalobiaceae* as the main bacterial family (Figure 1). They have been already reported to be present in high abundances in oil and gas reservoirs (Tinker et al., 2020), hypersaline environments as the Great Salt Lake (Kjeldsen et al., 2007), and salt caverns (Schwab et al., 2022). They were also found in acidic environments with high concentrations of iron and arsenic (Giloteaux et al., 2013), being involved in sulfate and thiosulfate reduction, and biofilm production (Tinker et al., 2020). Representatives, like the species *Desulfovermiculus halophilus*, are capable of growing in a salt concentration of up to 230 g L^–1^ using H_2_/CO_2_ for sulfate reduction (Beliakova et al., 2006). The detection of sulfate-reducing and autotrophic *Desulfohalobiaceae* next to halophilic archaea in this environment can be correlated with co-dissolution of sulfate-anions from the Zechstein formation. In fact, a coexistence, potential symbiosis, synergistic metabolism and magnificent influence in the carbon cycle of archaea and *Desulfobacteria* were already observed and hypothesized before (Zhao and Biddle, 2021). It would be worth to test, whether halophilic *Desulfobacteria* are astrobiological relevant candidates capable of surviving extraterrestrial conditions, including higher levels of radiation or desiccation.

Although *Salinibacter* was reported to be the most prominent bacterial genus within saline ecosystems, accounting for 5%–25% of the total prokaryotic community (Antón et al., 2000), only 0.29% and 0.22% of the reads were attributed here to this genus, revealing a difference in the microbial community associated with the ancient Permian formations compared to other saline ecosystems. This finding aligns with other studies, that do not classify *Salinibacter* as part of the core microbial community within the Zechstein formation (Schwab et al., 2022).

Interestingly, major parts of the amplicon sequences were not able to be assigned to any phyla, leading to the assumption that the Lunenburg brine may harbor yet unknown and uncultivated microorganisms (Figure 1, Supplementary Tables 3, 4). A substantial number of reads were assigned or related to the yet-uncultivated phyla *Patescibacteria* (also known as Candidate Phyla Radiation (CPR), historically referred to diffferent labels, e.g., OP11/OD1/WWE3), which members are ultra-small bacteria (∼0.009 μm^3^), known to have reduced genomes, and being metabolically dependent on other community microorganisms (Luef et al., 2015). In fact, it was recently discovered that the Candidatus *Patescibacteria* may also form a symbiosis with methanogenic archaea (Kuroda et al., 2022). They were already found to be prevalent in aquifer environments including groundwaters (Tian et al., 2020), spanning both fully oxidized and anoxic conditions (Chaudhari et al., 2021) as well as wastewaters with increased salinity and osmotic stress (Zhang et al., 2025). Researchers hypothesized that the majority of these microorganisms depend on forming non-specific attachments to various hosts to support their metabolic functions (Chaudhari et al., 2021). Even though most genomes of *Patescibacteria* were retrieved from groundwater ecosystems, they are also present in for example lakes (Chiriac et al., 2022), and as our study shows, also highly represented in extreme saline environments, expanding the environmental range for these yet-uncultivated microorganisms. Although microbial diversity in extraterrestrial hypersaline brines will likely differ substantially, analyzing key microbial taxa in terrestrial hypersaline brines can illuminate potential metabolic pathways in those extraterrestrial environments.

#### 4.3.2 RNA operons variation and osmotic adaptation within cultivated halophilic archaea

Two to three copies within the RNA operon of *Halorubrum* sp. AS12 and of *Haloarcula* sp. NS06 were detected and in case of NS06 distributed across two different replicons (Supplementary Tables 5–7), as already reported in another study investigating the *Haloarcula marismortui* genome (Baliga et al., 2004). Multiple copies of metabolically important genes may enhance survival in halite by providing protection against irreparable DNA damage, which can result from the significant levels of radiation reported in halite environments (Kminek et al., 2003).

In addition, 16S rRNA sequence analysis of NS06 lead to a 5% divergence among the two different copies within the primary chromosome (Supplementary Table 8). In fact, differences in the 16S rRNA gene up to 5%–9% were already reported before within the halophilic archaea *Haloarcula* and *Halomicrobium* (Cui et al., 2009; Dennis et al., 1998). Hence, whole genome sequencing, targeting other highly conserved genes, as well as proteotyping might reveal a good alternative for those candidates as already suggested (Runzheimer et al., 2024). Depending on the conserved gene analyzed, NS06 was assigned to several different *Haloarcula* species. In contrast, AS12 was predominantly associated with *Halorubrum ruber* MBLA099^T^, suggesting a potentially closer relationship to this species compared to the 16S rRNA-based identification with *Halorubrum terrestre* JCM 10247^T^ (Supplementary Table 8).

Different copies of the 16S rRNA gene within haloarchaea were already described to be beneficial within higher cultivation temperatures. A study by Sato and Kimura revealed that higher temperatures lead to higher expression of the 16S rRNA gene with higher GC content (Sato and Kimura, 2019). Further research on the expression of the different 16S rRNA copies under different stress conditions would hint on potential adaptation mechanisms of haloarchaea, since this divergence is especially, even though not exclusively, known among halophilic microorganisms.

A partial 109-nucleotide insertion related to 5.8S rRNA was detected in *Haloarcula* sp. NS06, showing 100% similarity across all rRNA operons (Supplementary Tables 5, 6). Large extensions within rRNA are rare among archaea and have previously been described only in the *Halobacteriales* order including *Halococcus* (Luehrsen et al., 1981; Tirumalai et al., 2020; Stepanov and Fox, 2021) and ASGARD archaea (Penev et al., 2020). Although this finding had not previously been reported for the genus *Haloarcula*, further analysis confirmed that it had already been sequenced. Further research on expansions within the 5S rRNA would be a valuable direction for future research on evolutionary steps, horizontal transfers or functional stability.

Both strains exhibit a characteristic halophilic proteome signature: an overall acidic proteome with an average pI around 4.9 (Supplementary Tables 6, 7). This aligns with previous research investigating the pI of *Haloarcula* and *Natrinema* with a major peak around 4 (Straková et al., 2025) and around 4.5 for *Haloferax mediterranei*, *Halorubrum litoreum*, *Haloarcula argentinensis*, and *Natrialba aegyptia* (Becker et al., 2014). The low average pI of haloarchaea reflects the predominance of acidic proteins, which enhances the hydration of the proteins’ shells, preventing “salting-out” and thus maintain enzymatic function in highly saline environments (Hartman et al., 2010). The extremely acidic hypothetical proteins identified within *Halorubrum* sp. AS12 and *Haloarcula* sp. NS06 with a pI below 3 may represent novel adaptation-related factors or annotation artifacts.

The distinct halophilic adaptations of *Halorubrum* sp. AS12 and *Haloarcula* sp. NS06 observed (Supplementary Table 9), reflect the differences in genome size and plasmid count (Supplementary Table 5). The genome of NS06 is more than 1 Mbp larger and contains multiple plasmids, whereas AS12 has only one single plasmid. Major differences in regard to halophilic adaptation include the absence of halorhodopsin in AS12 and an overall higher abundance of osmoadaptation genes in NS06. Both microorganisms encode multiple antiporter systems for Na^+^, K^+^ and Mg^2+^ including genes related to the antiporter *NhaP2*, *NhaC*, *Kef*, and *Trk*, *KdpABC* transporter as well as mechanosensitive channels. The *NhaP* family is essential for Na^+^/H^+^ exchange to control cytoplasmic pH, volume homeostasis, and intracellular Na^+^ levels (Resch et al., 2011), since elevated Na^+^ can inhibit enzyme activity (Brown and Simpson, 1972). *NhaP2* specifically can also function as a K^+^/H^+^ antiporter as shown already for *Vibrio cholerae* (Resch et al., 2010). The *Trk* system actively imports K^+^, which is crucial for salt-stress resilience (Corratgé-Faillie et al., 2010). To offset the positive charge from K^+^ uptake, cells co-import Cl^+^ via the light-driven chloride pump halorhodopsin (Schobert and Lanyi, 1982; Straková et al., 2025). However, as mentioned before, it was absent in *Haloarcula* sp. AS12. While *NhaC, Kef*, mechanosensitive channel, and the *Trk* system are generally encoded within halophilic archaea, the presence of *KdpABC* and *CorA* is not universal for haloarchaea (Becker et al., 2014), though being present in both isolates (*KdpB*) or at least in NS06 (*CorA*). Overall, *Haloarcula* sp. NS06 displays a broader genetic repertoire for ion transport, suggesting more effective salt adaptation than AS12, which is closely related to the greater genome size of NS06.

In terms of compatible solutes, both isolates possess genes for trehalose metabolism and glycine-betaine synthesis, osmolytes known to predominate in halophilic archaea (Youssef et al., 2013). Within the study of Youssef et al. (2013), *OtsA/B* genes, encoding for trehalose-6-phosphate synthase/phosphatase, were incomplete in analyzed *Halorubrum* and *Haloarcula* strains. However, in our annotations *OtsA/B* genes (*TpsP*) were also present within the *Haloarcula* sp. NS06 isolate. In contrast, *Halorubrum* sp. AS12 relies on the enzyme treT and is synthesizing trehalose via the trehalose glycosyl-transferring synthase pathway. Even though, parts of ectoine synthesis were reported before for the genus *Haloarcula* (Straková et al., 2025), we could not detect any related genes. Measurements of the intracellular concentrations of compatible solutes, particularly proline, revealed higher levels in *Halorubrum* sp. AS12 than in *Haloarcula* sp. NS06, although absolute amounts were generally low (< 0.1 fmol cell^–1^) (Supplementary Table 10). The comparatively high proline concentration (0.02 fmol cell^−1^) is consistent with the presence of the complete proline biosynthesis pathway in AS12 (*proA, proB, proC*), which is absent in NS06 (Supplementary Table 9).

Low average pI and comparably low measured intracellular concentrations of compatible solutes support osmoprotection via the “salt-in” strategy. For instance, ectoine concentrations from 1.7 to 142 fmol cell^−1^ have been reported for different phytoplankton species. In the bacterium *Pelagibaca bermudensis*, ectoine peaked at 1.33 M NaCl (171 pmol μg^–1^ protein) and was 78-fold higher than at 0.17 M (Azizah and Pohnert, 2024). To fully assess the role of proline, glycine-betaine, sarcosine, choline, and ectoine in the osmoprotection of *Halorubrum* sp. AS12 and *Haloarcula* sp. NS06, their intracellular concentrations under osmotic shock and at different growth rates need to be investigated.

### 4.4 Morphological variability

#### 4.4.1 Pleomorphism and cell aggregation

During the morphological investigation of two representative haloarchaea isolated from the brine: *Haloarcula* sp. NS06 and *Halorubrum* sp. AS12, we discovered that isolate NS06 demonstrated a variable morphology ranging from spherical to rod-shaped small cells present as single cells or short chains to larger aggregates of larger cells, which are enclosed by a fibrous sheath and which we termed “package-like aggregates” (Figure 2). For isolate *Halorubrum* sp. AS12, we observed a similar pleomorphism, with coccoid- and rod-shaped cells. However, we did not observe “package-like aggregates” as in isolate NS06 (Supplementary Figure 2).

Pleomorphism of the genera *Haloarcula* (Schwarzer et al., 2021) and *Haloferax* (de Silva et al., 2021) has already been observed before. For example, Schwarzer and colleagues reported a growth dependent cell shape for the species *Haloarcula hispanica* and *Haloarcula californiae.* According to the authors, *Haloarcula* cells tend to exhibit a rod-shaped morphology in the early exponentially phase, whereas in the stationary phase, cells appear to be a mixed population of rod-, round-, triangle- as well as a square-shaped cells. Thus, it is suggested that the morphology depends on motility, since in the early growth phase, motility is advantageous (Schwarzer et al., 2021). Recent findings have shown that mechanical pressure can trigger multicellular structures in *Haloferax volcanii* (Rados et al., 2025; Pillai and Brunet, 2025). Similarly, the *Haloarcula* strain studied here, demonstrated a form of “package-like aggregates”, which may represent an alternative strategy for environmental adaptation (Figures 2A, B).

Next to pleomorphism and cellular aggregation, bright inclusions within the cells of *Haloarcula* sp. NS06 were observed (Figure 2D). Inclusions within cells represent lipids, PHA/PHB accumulation or gas vesicles. Up to date, only a few halophilic microorganisms have been observed to produce gas vesicles. This includes members of halophilic microorganisms like anaerobic endospore forming bacteria, as well as the halophilic archaeal family *Halobacteriaceae*. Only 5.8% of species from this family originating from the genera *Halobacterium*, *Haloquadratum*, *Halorubrum*, *Halogeometricum*, *Haloferax*, and *Haloplanus* are able to produce gas vesicles (Oren, 2013). However, production of gas vesicles within *Haloarcula* species were not reported before. It is believed, that gas vesicles enable cells to reach the brine surface waters and thus stay in touch with atmospheric oxygen [reviewed by Singh and Singh (2017)]. At the initial and exponential growth stage, motility in halophilic archaea is mediated by the ATP-dependent archaella (Li et al., 2019), but as nutrients become scarce or depleted, gas vesicles morphogenesis enables the cells to position themselves advantageously to the water surface as shown for the bacterium *Serratia* (Ramsay and Salmond, 2012). Increased light intensities at the surface may further be beneficial for light harvesting using the light-driven proton pump bacteriorhodopsin (Oren, 2013). In this study, we were able to document the genetic repertoire for bacteriorhodopsin production within the genome of NS06 and AS12, supporting this hypothesis (Supplementary Table 9). The arrangement of the bacteriorhodopsin gene within the V-ATPase operon appears to be conserved in haloarchaea, but is not universal (Verma et al., 2020). For NS06, the presence of bacteriorhodopsin in the genome inserted between the genes coding for B and D subunits of the V-type ATPases operon was confirmed within the Prokka Annotation (Supplementary Table 6), whereas the Prokka annotation of AS12 revealed only hypothetical proteins without evidence of bacteriorhodopsin and was only identified within the RefSeq annotation (Supplementary Tables 7, 9).

Further investigation of the vesicles using fluorescence microscopy and staining with the red, lipophilic dye Nile Red revealed that they contain lipid-rich structures (Figure 3). Significant amounts of the lipids polyhydroxyalkanoate (PHA), polyhydroxybutyrate (PHB) and polyhydroxyvalerate (PHV) have been reported in halophilic archaea [reviewed by Simó-Cabrera et al. (2021)]. It his study, the genes responsible for PHA production were identified in *Haloarcula* sp. NS06 (Supplementary Table 6), but not in *Halorubrum* sp. AS12 (Supplementary Table 7), despite a previous report of the presence of the *phaC* gene in the related species *Halorubrum lacusprofundi*. However, the similarity between the relevant protein (Hlac_1732) and the *phaC* subunit in *Halorubrum lacusprofundi* is limited to 47% sequence identity in the C-terminal region, suggesting only partial functional similarity (Williams et al., 2017). In contrast, we found no partial similarity between the genes within NS06 and those in the genome of AS12. Further research on genes involved in PHA/PHB production in *Halorubrum* is therefore needed.

Even though no spore-like stage is known for archaea (Radax et al., 2001), the aggregated and ensheathed form of the isolate NS06 with production of lipids might represent a specific morphology, enabling survival during starvation, which was described as spheres and persister cells before (Stan-Lotter and Fendrihan, 2015; Megaw and Gilmore, 2017).

#### 4.4.2 Carotenoid pigmentation

Within this study, Raman spectroscopy and genomic analysis revealed the presence of carotenoids, most likely bacterioruberin, within the two isolates *Halorubrum* sp. AS12 and *Haloarcula* sp. NS06 (Figure 4 and Supplementary Tables 6, 7). In general, most of halophilic archaea are known to produce pink pigments (Giani et al., 2024). Pigments are promising biomarkers for remote detection, e.g., with Raman spectroscopy on extraterrestrial bodies and may help to quickly identify potential life in outer space (Marshall et al., 2007). Building databases of spectra within microorganisms found on Earth will enable potential identification of such microorganisms (Marshall et al., 2007) in future missions like ExoMars Rosalind Franklin in 2028 (European Space Agency, 2023), which will be equipped with a Raman spectrometer (Rull et al., 2017).

The archaeal carotenoid bacterioruberin is suggested to protect from reactive oxygen species and improve the capability to survive higher radiation doses (Grivard et al., 2022; Kottemann et al., 2005). In fact, the isolate *Haloarcula* sp. NS06 revealed to withstand X-ray irradiation of > 1,000 Gy with less than one log reduction of the total cell count, while *Halorubrum* sp. AS12 revealed to be more adapted to desiccation than *Haloarcula* sp. NS06 (Runzheimer and Leuko, 2025). Pigmentation may also help cells to withstand natural ionizing radiation from the isotope ^40^K. Its cumulative dose becomes relevant over geological timescales and increases with K content and time in halite (Kminek et al., 2003). Although isolates from 250-million-year-old halite suggest that archaeal pigmentation can mitigate radiation damage, the age of the host salt does not determine the age of the microorganisms, further work is needed to constrain their provenance and timing.

## 5 Conclusion

This study provides the first examination of the microbial diversity found in the hypersaline brine of Lunenburg, a halite remnant of the Zechstein Sea located in northern Germany, and a potential terrestrial analogous site for brines in inner and outer space. The habitat is described by a near-NaCl-saturated brine harboring high amounts of sodium and chloride ions as well as traces of sulfate, potassium, magnesium, calcium and bromide, slightly acidic pH as well as low oxygen levels. With 16S rRNA amplicon sequencing, we predominantly identified the phyla *Desulfobacterota, Patescibacteria* and *Halobacterota* as a characterizing community. Several candidate phyla were identified, suggesting that the Lunenburg brine is an ideal habitat for further cultivation approaches of yet-uncultivated microorganisms like the most abundant archaeal family *Nanosalinaceae* from the *Nanohaloarchaeota* phylum. Targeted cultivation of haloarchaea enabled the isolation of representatives from the order *Halobacteriales*. Among them, we identified the pink pigment bacterioruberin and potential PHA accumulation within the cells in *Halorubrum* sp. AS12 and *Haloarcula* sp. NS06, while also identifying corresponding genes for PHA in *Haloarcula* sp. NS06. For *Haloarcula* sp. NS06 we identified cell aggregations, which we term “package-like aggregates”. Compared to *Halorubrum* sp. AS12, *Haloarcula* sp. NS06 possesses a wider array of genes related to osmotic adaptation, while halorhodopsin is missing from *Halorubrum* sp. AS12. Genes for synthesis of trehalose and glycine-betaine are present in both isolates. Further compatible solute analysis revealed overall low concentrations of glycine-betaine, 5-hydroxyectoine, gonyol, proline, ectoine, sarcosine, trigonelline, and choline in both isolates, with higher levels in *Halorubrum* sp. AS12 and a clear dominance of proline. Both genomes revealed a low average isoelectric point of the proteome. Low concentrations of compatible solutes and overall low isoelectric points support the prevalence of the “salt-in” strategy. For *Haloarcula* sp. NS06, we detected three copies of the 16S rRNA operon showing 5% divergence, while *Halorubrum* sp. AS12 carried two copies differing by only 1%. In summary, cultivated isolates may survived the halite conditions by production of pigments, PHA accumulation, a diverse osmotic adaption repertoire with a focus on the “salt-in” strategy, cell aggregation and multiple copies of metabolic relevant genes. The Lunenburg brine presents an extreme, ideal and easily accessible habitat for further extremophilic and astrobiological research and analysis of yet-uncultivated microorganisms.

## Data Availability

All 16S rRNA gene sequences have been deposited in the NCBI database (https://www.ncbi.nlm.nih.gov) under accession numbers PV344485-PV344489. Genome sequences are available under the BioProject numbers PRJNA1236223 (*Halorubrum* sp. AS12) and PRJNA1236224 (*Haloarcula* sp. NS06) in the NCBI database. Raw reads from 16S rRNA amplicon sequencing are available within the Bioproject numbers PRJNA1303425 (V1/V2 and V3/V4) PRJNA1311456 (enrichments) in NCBI.

## Supplementary material

Supplementary Figure 1 Amplicon sequencing results of the enrichment cultures of the Lunenburg brine.

Supplementary Figure 2 Further microscopic information on *Halorubrum* sp. AS12.

Supplementary Table 1 Supplementary information regarding cultivated isolates and Sanger sequencing identification.

Supplementary Table 2 General information on the amplicon sequencing run of the environmental DNA from Lunenburg brine.

Supplementary Table 3 Top 10 abundant phyla and families detected within V1/V2 and V3/V4 sequencing analysis.

Supplementary Table 4 Most frequent detected ASVs within environmental amplicon sequencing.

Supplementary Table 5 Whole genome sequencing details on two selected isolates namely *Halorubrum* sp. AS12 and *Haloarcula* sp. NS06.

Supplementary Table 6 Genomic annotation and isoelectric points (ip) of *Haloarcula* sp. NS06 determined by Prokka.

Supplementary Table 7 Genomic annotation and isoelectric points (ip) of *Halorubrum* sp. AS12 determined by Prokka.

Supplementary Table 8 Sequences of selected housekeeping genes of *Halorubrum* sp. AS12 and *Haloarcula* sp. NS06 and the next related identified organism.

Supplementary Table 9 Genes identified within the genomes involved in osmotic adaption of *Halorubrum* sp. AS12 and *Haloarcula* sp. NS06.

Supplementary Table 10 Measured compatible solutes within *Halorubrum* sp. AS12 and *Haloarcula* sp. NS06.
