# Supplementary material for: Life in the brine of Lunenburg, Germany: unveiling microorganisms associated with Zechstein salt deposits
Source: Front Microbiol. 2025 Nov 12;16:1625916. doi: 10.3389/fmicb.2025.1625916 (PMC12650772; doi:10.3389/fmicb.2025.1625916)
Supplement: Supplementary Figure 1 — Amplicon sequencing results of the enrichment cultures of the Lunenburg brine. [file Data_Sheet_1.pdf]

Supplementary Figures

Figure S1: Relative sequence abundance from enrichment cultures at the genus level. Media compositions were R2A medium (HiMedia) at 3.12 g L^{-1} prepared in 100% brine, a 1:1 (v/v) mixture of brine and R2A (1.56 g L^{-1}), artificial seawater (ASW), Marine Broth (Difco) adjusted to 195 g L^{-1} NaCl, and brine supplemented with 0.1% (w/v) yeast extract and 0.075% (w/v) casamino acids.

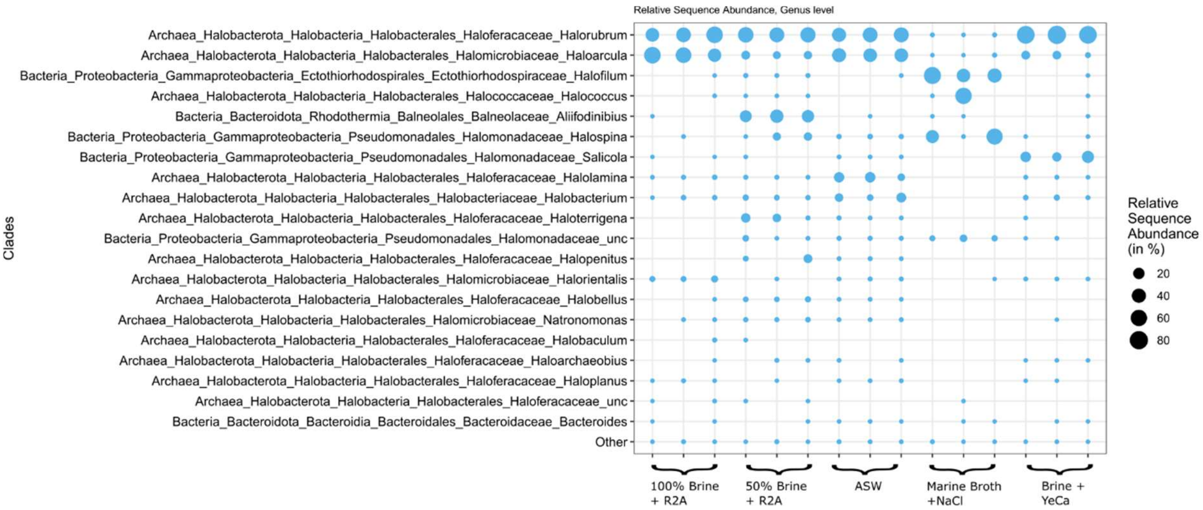

Figure S2: Microscopic morphology of *Halorubrum* sp. AS12 in light (A), thin section (B) and scanning-electron microscopy (C, D). (A) Single cells in a 21-days-old ASW suspension culture are small and almost coccoidal in shape. (B) Transmission-electron microscopy of a thin section through a colony which was grown for one week on a polyester filter placed on ASW agar. The image shows section profiles of three different cells, which are thin rods or coccoidal in shape (upper profile). The cytoplasm appears dense and granular. One profile shows a circular region of lesser density (arrowhead). The cell is limited by a thin and dense layer which is separated from the plasma by a bright appearing gap most probably representing the central part of the plasma membrane. Note, that the cells are connected by fine fibrous material (arrows). (C, D) Scanning-electron microscopy of colonies grown directly on ASW agar for a week. Cells are pleomorphic and differ in size and they are interconnected by a complex matrix (*). Scale bars represent 5 µm (A), 0.2 µm (B), 2 µm (C) and 1 µm (D).

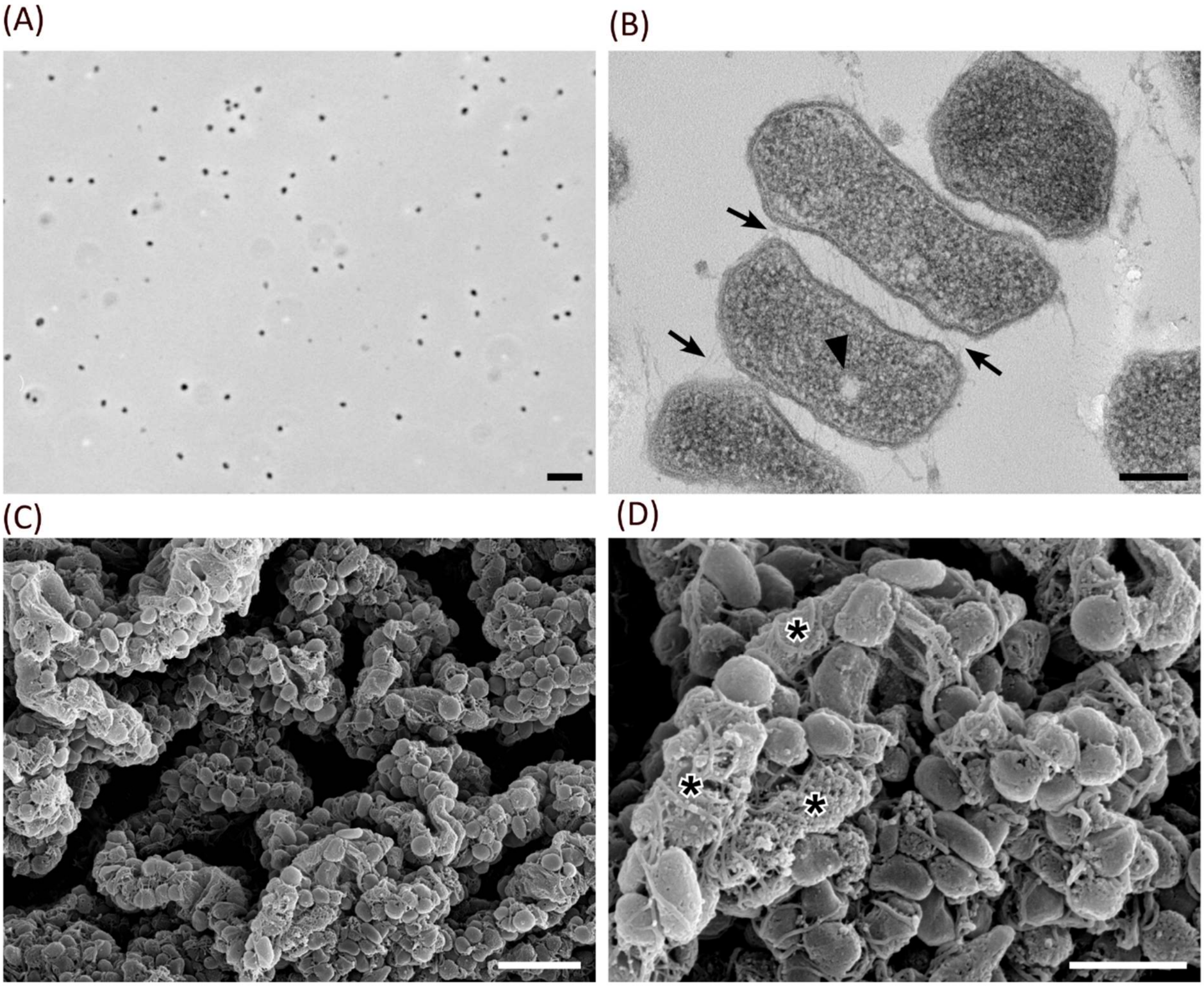
(A)
(B)
(C)
(D)
